# A cross‐scale analysis to understand and quantify the effects of photosynthetic enhancement on crop growth and yield across environments

**DOI:** 10.1111/pce.14453

**Published:** 2022-10-20

**Authors:** Alex Wu, Jason Brider, Florian A. Busch, Min Chen, Karine Chenu, Victoria C. Clarke, Brian Collins, Maria Ermakova, John R. Evans, Graham D. Farquhar, Britta Forster, Robert T. Furbank, Michael Groszmann, Miguel A. Hernandez‐Prieto, Benedict M. Long, Greg Mclean, Andries Potgieter, G. Dean Price, Robert E. Sharwood, Michael Stower, Erik van Oosterom, Susanne von Caemmerer, Spencer M. Whitney, Graeme L. Hammer

**Affiliations:** ^1^ ARC Centre of Excellence for Translational Photosynthesis, Centre for Crop Science, Queensland Alliance for Agriculture and Food Innovation The University of Queensland Brisbane Queensland Australia; ^2^ ARC Centre of Excellence for Translational Photosynthesis, Division of Plant Science, Research School of Biology The Australian National University Canberra Australian Capital Territory Australia; ^3^ School of Biosciences University of Birmingham Birmingham UK; ^4^ Birmingham Institute of Forest Research University of Birmingham Birmingham UK; ^5^ ARC Centre of Excellence for Translational Photosynthesis, School of Life and Environmental Science, Faculty of Science University of Sydney Sydney New South Wales Australia; ^6^ College of Science and Engineering James Cook University Townsville Queensland Australia; ^7^ Hawkesbury Institute for the Environment Western Sydney University Richmond New South Wales Australia

**Keywords:** APSIM, crop growth modelling, crop production, cross‐scale model, electron transport‐limited photosynthesis, enzyme‐limited photosynthesis, yield improvement

## Abstract

Photosynthetic manipulation provides new opportunities for enhancing crop yield. However, understanding and quantifying the importance of individual and multiple manipulations on the seasonal biomass growth and yield performance of target crops across variable production environments is limited. Using a state‐of‐the‐art cross‐scale model in the APSIM platform we predicted the impact of altering photosynthesis on the enzyme‐limited (*A*_c_) and electron transport‐limited (*A*_j_) rates, seasonal dynamics in canopy photosynthesis, biomass growth, and yield formation via large multiyear‐by‐location crop growth simulations. A broad list of promising strategies to improve photosynthesis for C_3_ wheat and C_4_ sorghum were simulated. In the top decile of seasonal outcomes, yield gains were predicted to be modest, ranging between 0% and 8%, depending on the manipulation and crop type. We report how photosynthetic enhancement can affect the timing and severity of water and nitrogen stress on the growing crop, resulting in nonintuitive seasonal crop dynamics and yield outcomes. We predicted that strategies enhancing *A*_c_ alone generate more consistent but smaller yield gains across all water and nitrogen environments, *A*_j_ enhancement alone generates larger gains but is undesirable in more marginal environments. Large increases in both *A*_c_ and *A*_j_ generate the highest gains across all environments. Yield outcomes of the tested manipulation strategies were predicted and compared for realistic Australian wheat and sorghum production. This study uniquely unpacks complex cross‐scale interactions between photosynthesis and seasonal crop dynamics and improves understanding and quantification of the potential impact of photosynthesis traits (or lack of it) for crop improvement research.

## Summary Statement

Grain yield per area information of photosynthetically manipulated crop plants is limited. Cross‐scale modelling predicted how growth dynamics of wheat and sorghum crops can be affected and quantified various manipulation impacts on grain yield in multiple environments for crop improvement research.

## 1 INTRODUCTION

New strategies to improve grain yield in globally important staple crops are needed urgently if production is to keep pace with growing demand (Fischer et al., 2014; Ray et al., 2013). Improving crop resource use efficiencies and crop growth rates are promising avenues and photosynthesis has emerged as one of the major traits of interest (Evans, 2013; Hammer et al., 2020; Long et al., 2015; Sharwood et al., 2022; von Caemmerer & Furbank, 2016). The feasibility of enhancing leaf CO_2_ assimilation rate has been demonstrated in many transgenic studies (e.g., Ermakova et al., 2019; Salesse‐Smith et al., 2018). There is also evidence of enhanced single‐plant biomass and/or seed weight in cereal crop species (Simkin et al., 2019). Field experiments with free‐air CO_2_ enrichment studies and transgenic model species provide further empirical evidence of the potential for crop improvement under non‐stressed conditions (Ainsworth & Long, 2021; South et al., 2019). Despite efforts in leaf photosynthetic engineering and pot/field studies, knowledge of how manipulations can influence yield performance in target crops grown across multiple environments is limited (Fischer et al., 1998). Others have suggested photosynthesis only has a minor role in determining crop yield when scaling from leaf photosynthetic rate to grain yield (Sinclair et al., 2019). The conflicting evidence heightened the need for research to better understand nonintuitive crop‐by‐environment interactions and quantify emergent yield outcomes, either positive or negative, arising from manipulating photosynthetic traits (Hammer et al., 2016).

Addressing the knowledge gaps requires an understanding of interactions between perturbed leaf photosynthesis and crop biomass growth rates, whole‐plant developmental processes, crop carbon, water, and nitrogen uptake/allocation amongst organs, and feedback regulation on leaf photosynthesis by the status of the crop and the environment (Hammer et al., 2010; Wu et al., 2016). Furthermore, these need to be analysed in multiple environments representative of where the crops would be grown (i.e., target population of environments). Ideally, photosynthetically enhanced plants need to be tested using multi‐environment trials (i.e., field testing of target crops at several representative production locations over several years) for better understanding and quantification of growth and yield dynamics, but such an approach is mostly inaccessible. The absence of such information hampers efforts to maximize the rate of yield improvement (Fischer et al., 2014).

Crop growth modelling is a useful method for predicting the growth, development, and yield dynamics of the crop and their interactions with the growing environment over the crop life cycle. The interpretive and predictive potential of crop growth models enables simulation of consequences of trait manipulation across different environmental conditions and crop management practices (Hammer et al., 2019a). Recent research thrusts in crop growth modelling paved the way for achieving the necessary leaf‐to‐crop connection for analysing leaf photosynthetic manipulation (Chew et al., 2017; Hammer et al., 2019b; Marshall‐Colon et al., 2017; Wu et al., 2016). They proposed combining models across multiple scales of biological organisations to predict emergent cross‐scale effects. This involves models that incorporate complexities associated with interactions between leaf photosynthetic rates, diurnally changing temperature, solar radiation, and within canopy light environment to predict daily canopy photosynthetic and/or crop growth rates (e.g., de Pury & Farquhar, 1997; Hammer & Wright, 1994; Song et al., 2013; Wu et al., 2018). This can be connected with crop models that incorporate complex interactions between crop phenology, canopy development, growth, and effects of whole‐crop water and nitrogen supply/demand on growth and development processes (Brown et al., 2014; Hammer et al., 2010) to provide feedback input for predicting leaf and canopy photosynthesis over the crop life cycle.

**Figure 1:**
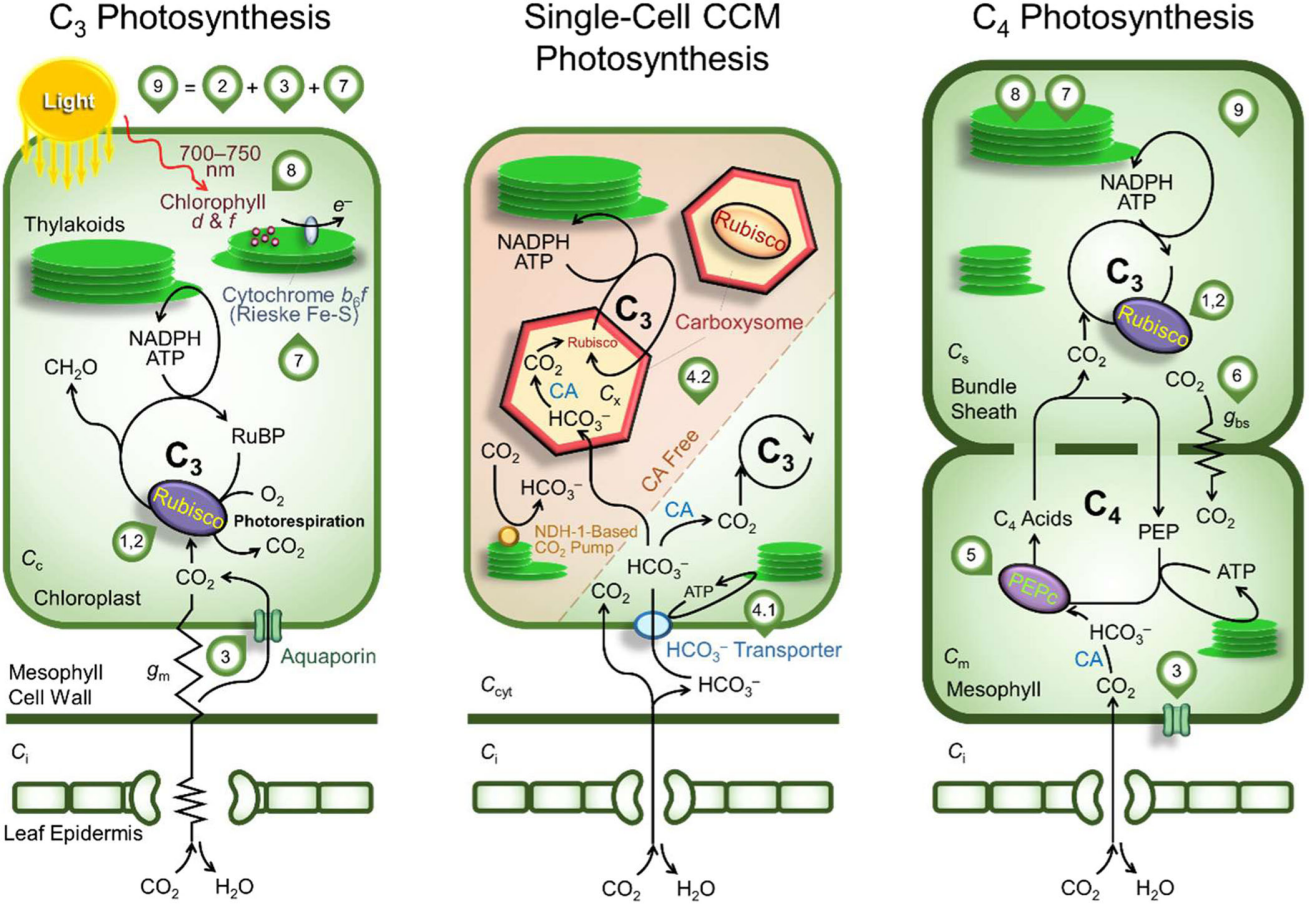
Overview of the leaf photosynthetic pathways and manipulation targets used in wheat and sorghum crop growth and yield simulations. The manipulation targets are numbered here and detailed in Table 1. Bioengineering strategy ‘1, 2’ encompasses ‘1.1’, ‘1.2’, ‘1.3’, ‘1.4’ and ‘2’. Strategy ‘9’ is achieved by stacking ‘2’, ‘3’ and ‘7’. Graphics for stomatal and mesophyll resistance/conductance are omitted in the single‐cell CCM and C_4_ photosynthesis pathways for simplicity. *C*_i_, *C*_c_, *C*_m_, C_x_ and *C*_s_ are the intercellular, chloroplastic, mesophyll, carboxysomal and bundle sheath CO_2_ partial pressures; CA, carbonic anhydrase; *g*_bs_, bundle sheath conductance; *g*_m_, mesophyll conductance.

The state‐of‐the‐art cross‐scale modelling capability has been used in a number of studies to predict grain yield outcome of wheat and sorghum in single production environment simulation by varying photosynthetic model parameter values or simulating manipulation outcomes observed at the leaf level (Hammer et al., 2019b; Wu et al., 2019). Others have predicted rice biomass growth outcomes under well‐watered and fertilized conditions (Yin & Struik, 2017). Recent progress in photosynthetic engineering studies has advanced leaf‐level knowledge relating to Rubisco function, electron transport chain, CO_2_ delivery, and manipulation stacking (see Figure 1 and references in Table 1). Using the interpretive and predictive natures of the cross‐scale model, it is possible to generate new diagnostic analyses relating to how much photosynthetic manipulation can drive and be regulated by seasonal crop biomass growth and yield dynamics. In addition, given the prevalence of water and nitrogen stress across global crop production, there is a need to improve the understanding of contrasting water and nitrogen effects across multiple production environments. Models such as the cross‐scale model in the APSIM platform that have been demonstrated to predict field crop data in a wide range of environments by capturing the two‐way interactions between leaf photosynthesis and crop growth and yield processes will be invaluable (Wu et al., 2016, 2019).

**Table 1:** Comprehensive information on modelling effects of photosynthetic manipulation at the leaf level. This includes key aspects of leaf photosynthetic function in C_3_ wheat and C_4_ sorghum, the associated manipulation outcomes, bioengineering strategies, and representation using the generic leaf photosynthesis model coupled with CO_2_ diffusion processes (Supporting Information: Appendix A). Component(s) of the photosynthetic machinery relevant to the bioengineering strategies are indicated in the schematic diagram of photosynthetic pathways (Figure 1). The relative changes in the photosynthetic parameters are applied to the baseline set (Supporting Information: Table S5).

Key aspects of leaf photosynthetic function	Manipulation outcome	Bioengineering strategy	Modelling of bioengineering targets with the generic leaf photosynthesis model
Rubisco function	(1.1) Rubisco with desirable C_4_ NADP‐ME maize catalytic properties (in C_3_ wheat)	Engineering the endogenous Rubisco to achieve increased $k_{\mathrm{cat}}^{c}$ and carboxylation efficiency $\left(\text{CE:}k_{\text{cat}}^{c}/\left[K_{c}\left(1+\frac{O}{K_{o}}\right)\right]\right)$ as found in C_4_ maize Rubisco.	+50% $k_{\text{cat}}^{c}$, but only a +30% CE (as a result of concomitant changes in *K*_c_ (+18%) and *K*_o_ (+3%)) (Sharwood et al., 2016a, 2016b); assumed no change in *S*_c/o_ or temperature response of any of the Rubisco catalytic properties; $k_{\text{cat}}^{c}$ increase is modelled by a proportional 50% increase in *V*_cmax_ assuming the amount of Rubisco enzyme is the same and no change in the activation state.
	(1.2) Reducing wasteful photorespiration with higher specificity for CO_2_ over O_2_ (*S*_c/o_) (in C_3_ wheat)	Engineering the endogenous Rubisco to increase *S*_c/o_.	+20% *S*_c/o_ (Martin‐Avila et al., 2020); assumed no change in *V*_cmax_, *K*_c_ and *K*_o_ or temperature response or any of the Rubisco catalytic properties.
	(1.3) Increased Rubisco content (in C_4_ sorghum)	Overexpressing the endogenous Rubisco.	+20% *V*_cmax_ (Salesse‐Smith et al., 2018).
	(1.4) Reduced affinity of Rubisco for oxygen (in C_4_ sorghum)	Engineering the endogenous Rubisco to increase *K*_o_ (von Caemmerer & Furbank, 2016).	+20% increase in *K*_o_; concomitant change in *S*_c/o_; assumed no change in *V*_cmax_ and *K*_c_ or temperature response of any of the Rubisco catalytic properties.
	(2) ‘Better’ Rubisco:	C_3_ wheat:	$k_{\text{cat}}^{c}$ (or *V*_cmax_), CE, and *S*_c/o_ modified as described for Targets 1.1 and 1.2.
	C_3_: Rubisco having C_4_ NADP‐ME maize‐like catalytic properties and higher *S*_c/o_	Stacking manipulation in $k_{\text{cat}}^{c}$, CE and *S*_c/o_ from combining Targets 1.1 and 1.2.	
	C_4_: Increased amount and improved catalytic properties	C_4_ sorghum:	*V*_cmax_ and *K*_o_ (affecting *S*_c/o_) modified as described for Targets 1.3 and 1.4.
		Stacking manipulation in Rubisco amount and *S*_c/o_ from combining Targets 1.3 and 1.4.	
CO_2_ delivery	(3) Improved diffusion of CO_2_ into the mesophyll	Altering membrane permeability to CO_2_ by adding CO_2_ permeable plasma membrane intrinsic proteins, and aquaporins to increase mesophyll conductance (Groszmann et al., 2017).	+20% mesophyll conductance (*g*_m_) (Groszmann et al., 2017); assumed no change to the temperature response of *g*_m_.
	(4.1) Cyanobacterial CCM: active transport of dissolved inorganic carbon to reduce the drawdown of CO_2_ in the mesophyll (in C_3_ wheat)	Adding cyanobacterial ${\text{HCO}}_{3}^{-}$ transporters (single‐subunit BicA and SbtA) to the chloroplast envelope (Price et al., 2013).	A biochemical model of single‐cell CCM photosynthesis is derived by combining the single‐cell C_4_ model (von Caemmerer, 2003) and the C_4_ photosynthesis models (von Caemmerer, 2000) for modelling the cyanobacterium CCM in C_3_. Effects of adding cyanobacterial ${\text{HCO}}_{3}^{-}$ transporters are captured by:
			Additional cost of 0.75 ATP for active transport of ${\text{HCO}}_{3}^{-}$ into the mesophyll (Price et al., 2011), which equates to 20% of the total ATP consumption (including the 3 ATP required by the C_3_ cycle), to operate the two transporters; the ${\text{HCO}}_{3}^{-}$ transport rate with respect to CO_2_ levels is aggregated in a single set of maximal activity (*V*_bmax_) and Michaelis‐Menten constant (*K*_b_ = 60); *V*_bmax_ is assumed to scale linearly with leaf nitrogen content above the leaf structural N requirement for photosynthesis (slope = 0.36, which gives *V*_bmax_ of 45 μmol m^–2^ s^–1^ with wheat leaf SLN of 2 g N m^–2^;
			Induction of cyclic electron flow: in C_4_ photosynthesis, in addition to the requirement by the C_3_ cycle, the additional ATP costs in the mesophyll (*φ*) induces cyclic electron flow. The same effect is modelled for CCM in C_3_ by incorporating a multiplier *z* ($=\frac{3-f_{c\mathrm{yc}}}{4\left(1-f_{\mathrm{cyc}}\right)}$) to the linear electron flow (*J*), where *f*_cyc_ is the fraction of *J* out of PSI that proceeds via cyclic electron flow (von Caemmerer, 2021). This fraction is 0.25*φ* given that C_4_ with the additional 2 ATP requirement has *f*_cyc_ of 0.5 and C_3_ with no additional ATP requirement has negligible *f*_cyc_. The multiplier *z* is calculated to be 0.75 when *φ* is negligible as in C_3_ photosynthesis and is ca. 0.87 when *φ* = 0.75 for operating the transporters.
			The original mesophyll conductance is modelled by the cell wall plasmalemma interface and chloroplast envelope conductance in series. They are assumed 1 and 1 mol m^–2^ s^–1^ bar^–1^ (giving an intercellular–chloroplastic conductance of 0.5 mol m^–2^ s^–1^ bar^–1^ with leaf SLN of 2 g N m^–2^ at 25°C, comparable to that observed in C_3_ wheat).
	(4.2) Cyanobacterial CCM: full implementation of the cyanobacterial CCM (in C_3_ wheat)	Building on Target 4.1, the full CCM implementation involves adding carboxysomes containing carboxysomal Rubisco, and systems to further minimize CO_2_ diffusion from the site of carboxylation, which are likely to involve eliminating carbonic anhydrase from the chloroplast stroma, adding NDH‐1‐based CO_2_ pump, and/or making the chloroplast envelope less conductive to CO_2_ via aquaporin manipulation (Price et al., 2013).	The single‐cell CCM photosynthesis model is capable of simulating the full CCM implementation. In addition to those changes described in Target 4.1, the full CCM effects are modelled by:
			Replacing endogenous wheat Rubisco with a carboxysomal Rubisco. Kinetic properties of a carboxysomes‐encapsulated Rubisco with *K*_c_ increased by a factor of 9.9; *K*_o_ reduced by 17%, and *S*_c/o_ is reduced by 39%; derived from comparing Rubisco kinetics reported for wheat (Sharwood et al., 2016b) and *Cyanobium* Rubisco produced in transgenic tobacco plants (Long et al., 2018); assuming carboxylation and oxygenation temperature responses remain similar.
			Chloroplast envelope conductance is expected to reduce to levels similar to that in C_4_ species (i.e., 3 mmol m^–2^ s^–1^) (von Caemmerer, 2000)) with the installation of carboxysomes (having the desirable properties of retarding CO_2_ leakage and negligible effects on entry and exit of ${\text{HCO}}_{3}^{-}$) together with the additional biochemical changes regarding carbonic anhydrase and NDH‐1‐based CO_2_ pump.
			Assuming no change in *V*_cmax_ and photosynthetic machinery N requirement with Rubisco replacement and carboxysome synthesis. $k_{\text{cat}}^{c}$ of the encapsulated Rubisco could theoretically attain the levels of free *Cyanobium* Rubisco produced in the tobacco plants and those from *Cyanobium* cells (ca. 9.5 s^–1^), which is a factor of up to ca. 3 compared to those of wheat (Long et al., 2018). This means the same *V*_cmax_ can be achieved with less Rubisco, resulting in no additional N requirement or surplus N even after the costs of carboxysome are taken into account (Rae et al., 2017).
			Assuming efficient bicarbonate transport systems to achieve sufficiently high levels of chloroplastic bicarbonate concentrations. This is modelled by setting the maximal activity of the ${\text{HCO}}_{3}^{-}$ transporter (*V*_bmax_) at a level comparable to the maximal PEP carboxylase activity used in C_4_ sorghum (i.e., *V*_bmax_ of ~120 μmol CO_2_ m^–2^ s^–1^ with wheat leaf SLN of 2 g N m^–2^ at 25°C); if achieved by having more of both BicA and SbtA there would be no change in *K*_b_; temperature response of *V*_bmax_ and *K*_b_ are assumed same as the C_4_ equivalent in the initial carboxylation step.
	(5) Higher phosphoenolpyruvate carboxylation rate (in C_4_ sorghum)	Overexpressing PEP carboxylase (von Caemmerer & Furbank, 2016)	+20% maximum carboxylation rate of phosphoenolpyruvate (*V*_pmax_)
	(6) Reduced CO_2_ leakage from bundle sheath (in C_4_ sorghum)	Manipulating the interface between mesophyll and bundle sheath	–20% bundle sheath conductance for gases, affecting efflux of CO_2_ and O_2_ from the bundle sheath to the mesophyll, without affecting diffusive flux of the C_4_ and C_3_ cycle photosynthetic metabolites between the cells.
Electron transport chain	(7) More efficient electron transport chain	Overexpressing the Rieske FeS protein to generate more abundance of cytochrome b_6_f complex (Ermakova et al., 2019; Simkin et al., 2017).	Average of effects on parameters describing the *J*–*I* response inferred from photosynthetic light response data by (Ermakova et al., 2019; Simkin et al., 2017): +20% *J*_max_ (maximum electron flow at saturating light); +15% *J* response at low light; –40% empirical *J*–*I* curvature factor; assuming *J*_max_ has the same temperature response.
	(8) Access to additional light energy from photons in the 700–750 nm wavelength; increasing the fraction of PAR to total solar.	Swapping some chlorophyll *a* and *b* in Photosystems II and I with cyanobacterial Chlorophyll *d* and *f* in all leaves of the canopy (Chen & Blankenship, 2011).	+20% photosynthetic active radiation from solar radiation (both direct and diffuse) to all leaves in the canopy assuming Chlorophyll *d* and *f* can be constitutively expressed in all leaves and they are able to capture all photons in the 700–750 nm wavelength (Chen & Blankenship, 2011). Assumed no change to plant's ability to use the visible spectrum (400–700 nm); electron flow has the same light and temperature responses.
Manipulation stacking	(9) Combining ‘better’ Rubisco, higher electron transport rate, and improved diffusion of CO_2_ into the mesophyll	Combinations of the bioengineering approach described above.	Combining parameter changes described in Targets 2, 3 and 7.

The nonintuitive nature of cross‐scale effects and crop‐by‐environment interactions poses a great challenge for strategizing photosynthetic and crop improvement. Here, the current knowledge gaps in understanding the importance and quantifying the impact of photosynthesis traits in target cereal crops in multiple production environments were addressed through the following three objectives of this study: (1) compose a broad list of promising photosynthetic enhancement strategies for C_3_ and C_4_ photosynthesis relating to Rubisco function, electron transport chain, CO_2_ delivery, manipulation stacking, and predict their effects on the enzyme‐limited (*A*_c_) and electron transport‐limited (*A*_j_) rates of CO_2_ assimilation as a function of CO_2_ to show likely effects at the leaf level; (2) present unique leaf‐to‐crop diagnostic analysis using the cross‐scale crop growth model to unpack consequences of perturbed leaf photosynthesis on seasonal wheat and sorghum crop biomass growth and yield dynamics in contrasting water and nitrogen conditions in multiple production environments; (3) present a case study on national scale yield impacts for Australian wheat and sorghum production. Manipulation targets promising the greatest gains in crop production are identified.

## 2 MATERIALS AND METHODS

This modelling study aims to advance our understanding of how biomass growth and yield formation of C_3_ wheat and C_4_ sorghum crops respond to enhanced photosynthesis in realistic production environments. The state‐of‐the‐art cross‐scale model (Wu et al., 2019) used in this study mapped out the primary connections between key biochemical/physiological processes of leaf photosynthesis and stomatal conductance, which include the enzyme‐limited (*A*_c_) and electron transport‐limited (*A*_j_) rates of CO_2_ assimilation (Farquhar et al., 1980; von Caemmerer, 2000), photosynthesis at the canopy level using a sun–shade canopy approach (Wu et al., 2018), and crop growth, development, and yield processes captured in advanced wheat and sorghum crop models in the APSIM platform (Brown et al., 2014; Hammer et al., 2010; Holzworth et al., 2014). The cross‐scale model has previously been parameterized and extensively validated for predicting wheat and sorghum crop biomass growth and yield in contrasting water and nitrogen production environments (Wu et al., 2019). The interpretive and predictive capabilities of the model are used to understand the importance of photosynthesis traits and quantify their impacts on seasonal crop growth and yield dynamics. Below, we outlined baseline leaf and canopy photosynthesis modelling, followed by simulation of the crop cycle and multi‐environment setup, then introduce photosynthetic manipulation and modelling building on the baseline leaf, canopy, and whole‐crop simulation. Finally, we outlined the Australian crop production simulation setup.

### 2.1 Leaf and canopy photosynthesis simulation

For this modelling study, the C_3_/C_4_ leaf photosynthesis module coupled with CO_2_ diffusion processes and leaf energy balance calculations, previously incorporated in the cross‐scale model (Wu et al., 2019), was used. Briefly, the leaf‐level module is based on the biochemical models of C_3_ and C_4_ photosynthesis combined with a CO_2_ diffusion model based on Fick's law of diffusion that captures the diffusion of air CO_2_ to the site of Rubisco carboxylation. The C_3_ and C_4_ models followed those by Farquhar et al. (1980) and von Caemmerer (2000). The combined photosynthesis–CO_2_ diffusion model uses an input *C*_i_ for calculating leaf photosynthetic CO_2_ assimilation rate and stomatal conductance. These variables interact iteratively with leaf energy balance using the Penman–Monteith combination equation to calculate leaf transpiration and leaf temperature as set out by Wu et al. (2019).

Here, the previous photosynthesis–CO_2_ diffusion model was expanded to allow the modelling of a single‐cell design cyanobacterial CCM pathway. The CCM model followed Price et al. (2011). Briefly, it captures the active transport of dissolved inorganic carbon into the mesophyll and its take‐up by specialized protein micro‐compartments, carboxysomes, that concentrate CO_2_ around the encapsulated Rubisco (Price et al., 2013). The mesophyll and carboxysomes are modelled as two separate but connected, compartments with bicarbonate transporters driving the CO_2_ increase in the carboxysome and also accounting for CO_2_ leakage. The expanded generic photosynthesis–CO_2_ diffusion model has the capacity to simulate the effects of varying light, temperature, leaf nitrogen content, and transpiration on leaf CO_2_ assimilation rate (Wu et al., 2019) for C_3_, C_4_, and CCM photosynthetic pathways. A full description of the model and model equations is given in Supplementary Information: Appendix A.

The baseline set of the C_3_ and CCM wheat and C_4_ sorghum photosynthesis model parameters were adapted from Wu et al. (2019) with some recalculated using new data (Supplementary Information: Table S5). Key physiological parameters are the maximum rate of Rubisco carboxylation (*V*_cmax_), maximum rate of PEP carboxylation (*V*_pmax_), and maximum rate of electron transport at infinite light intensity (*J*_max_), and mesophyll conductance (*g*_m_). The baseline values of *V*_cmax25_ and *J*_max25_ for wheat (the subscripted number denotes value at the standard 25°C), and *V*_cmax25_, *V*_pmax25_ and *J*_max25_ for sorghum were set to those observed previously (Silva‐Pérez et al., 2017; Sonawane & Cousins, 2020; Sonawane et al., 2017). For the CCM pathway, the Michaelis–Menten constant for CO_2_ (converted from the constant for bicarbonate) (*K*_b_) (Price et al., 2011) was calculated from the CO_2_ response resulting from BicA and SbtA transporters combined. The maximum rate of bicarbonate transport (*V*_bmax_) was the sum of the BicA and SbtA transporters using values from Price et al. (2011).

The value of *J*_max25_ along with *α*_PSII_ and *θ* used in Supplementary Information: Equation S4 gave a potential whole‐chain linear electron transport rate (*J*) of 232 μmol m^–2^ s^–1^ at photosynthetic photon flux density (PPFD) of 1800 μmol m^–2^ s^–1^ and 25°C, comparable to that inferred from C_3_ wheat data (Silva‐Pérez et al., 2017). The ATP‐limited version of the electron transport‐limited equation was used in the single‐cell CCM model with a factor that relates *J* to the production of ATP (Supplementary Information: Equations S11 and S13) following von Caemmerer (2021). This treatment gave almost the same electron‐transport‐limited CO_2_ assimilation rate as the NADPH‐limited equation used in the C_3_ model (Supplementary Information: Equation S2). The C_4_ model also uses the ATP‐limited version of the equation. For the C_4_ electron transport parameters, the value of *J*_max25_ along with *α*_PSII_ and *θ* gave a *J* of 215 μmol m^–2^ s^–1^ at PPFD of 1800 μmol m^–2^ s^–1^ and 25°C comparable to that inferred from C_4_ maize data (Massad et al., 2007). The maximal activity of the bicarbonate transporters (*V*_bmax_) was taken from Price et al. (2011). In the full CCM case, a more efficient CO_2_ transportation rate comparable to that in the C_4_ version of the CCM was used as the system would require a higher inorganic carbon influx to function efficiently. If a low *V*_bmax_ was used, the yield would be significantly impacted due to reduced CO_2_ assimilation rate and growth (Supplementary Information: Figure S9).

**Figure 2:**
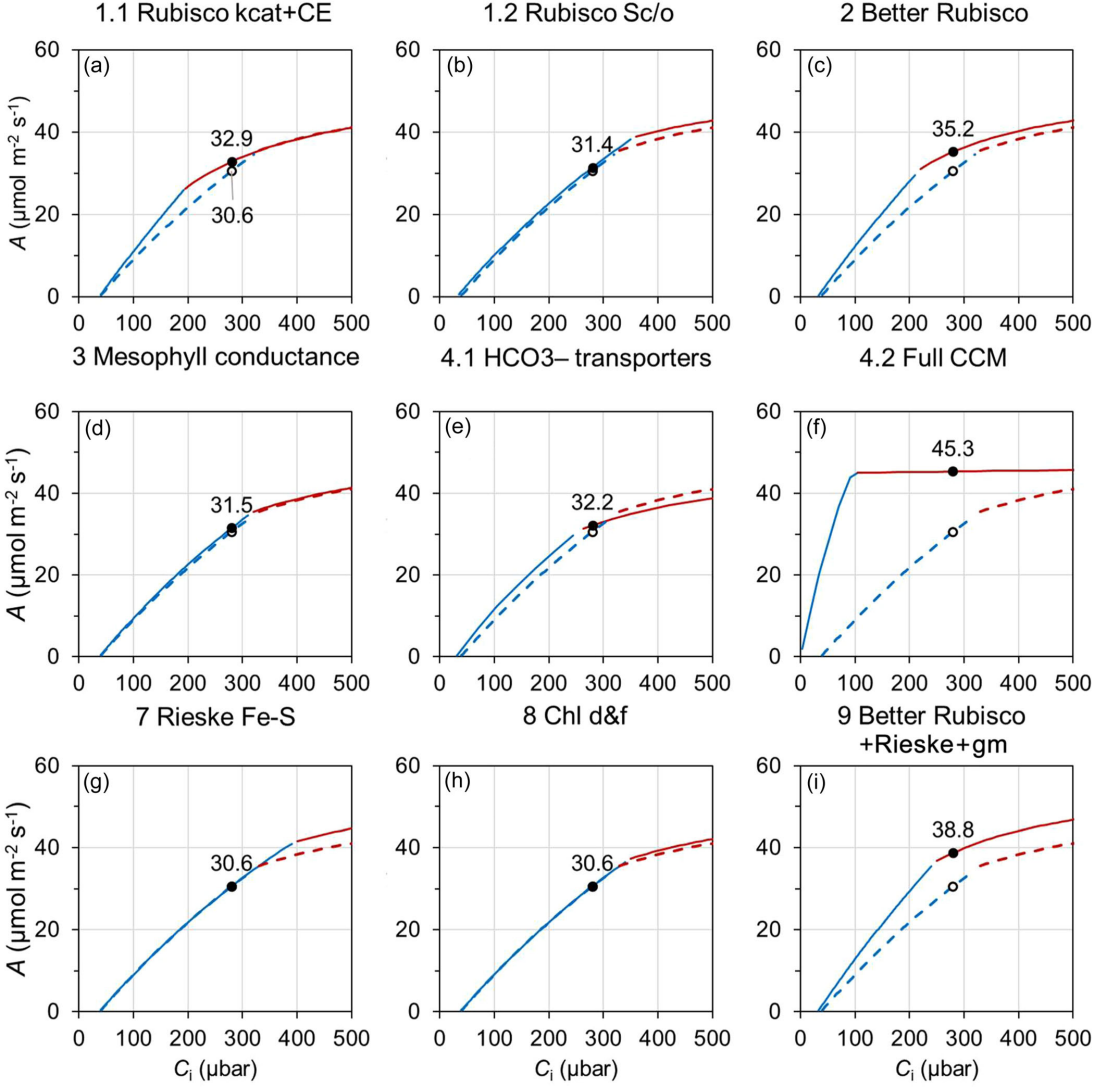
Simulated C_3_ wheat leaf photosynthetic response to intercellular CO_2_ (*A–C*_i_) for the baseline and manipulated scenarios. *A–C*_i_ are simulated for 25°C with photosynthetic photon flux density of 1800 μmol m^–2^ s^–1^ using the C_3_ and single‐cell CCM photosynthesis model parameter values given in Supporting Information: Table S5. Panels are for the different leaf photosynthetic manipulations as described in Table 1. The baseline *A–C*_i_ is reproduced in every panel as dashed lines; solid lines are *A–C*_i_ with photosynthetic manipulation. Blue and red are Rubisco activity (*A*_c_) and electron transport (*A*_j_) limited *A*, respectively. Unfilled and filled circles are *A* at an ambient CO_2_ of 400 μbar (i.e., intercellular CO_2_ of 280 μbar) for the baseline and with manipulations, respectively. The value of the baseline *A* is indicated in Panel (a); the manipulated *A* is given in all panels. (a–c) relate to Rubisco function manipulations, (d–f) relate to CO_2_ delivery manipulations, and (g–h) relate to electron transport chain manipulations, (i) a combination of the three aspects. Details of the manipulations are given in Table 1.

**Figure 3:**
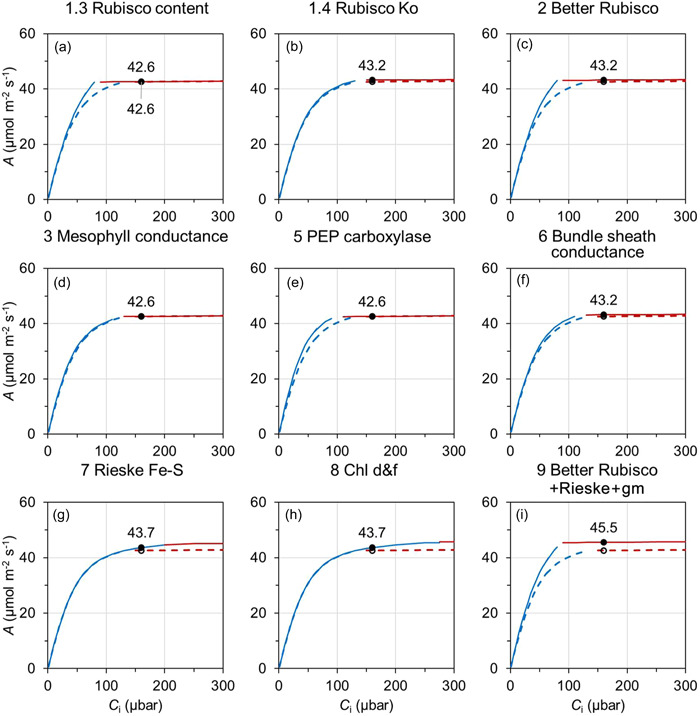
Simulated C_4_ sorghum leaf *A–C*_i_ for the baseline and manipulated scenarios. *A–C*_i_ is simulated for 30°C with photosynthetic photon flux density of 1800 μmol m^–2^ s^–1^ using the C_4_ sorghum photosynthesis model parameter values given in Supporting Information: Table S5. Lines and symbols are the same as those described in Figure 2.

The key physiological parameter (i.e., *V*_cmax25_, *J*_max25_, *V*_pmax25_, *V*_bmax25_ and *g*_m25_) values were used to calculate the corresponding *χ* values for input into the cross‐scale model, where each *χ* value is the slope of the linear relationship between the photosynthetic parameter and specific leaf nitrogen (SLN, g N m^–2^ leaf) (Supplementary Information: Table S5). The Rubisco catalytic properties and mesophyll conductance, the C_4_ bundle sheath conductance, and the baseline *C*_i_/*C*_a_ were taken from published data (Bernacchi et al., 2002; Boyd et al., 2015; Long et al., 2018; Massad et al., 2007; Ubierna et al., 2017; von Caemmerer & Evans, 2015) and a summary table by Wu et al. (2019). The photosynthetic parameters in Supplementary Information: Table S5 were used for simulating the baseline C_3_ and C_4_ *A*_c_ and *A*_j_ limitations, and *A*–*C*_i_ curves (Figures 2 and 3). The curves were comparable to those observed previously (Silva‐Pérez et al., 2017; Sonawane et al., 2017).

Leaf photosynthesis and stomatal conductance/transpiration were upscaled to the canopy level following Wu et al. (2019). Briefly, the leaf area of the canopy was partitioned into sunlit and shaded fractions (on a per‐ground area basis), and the key photosynthetic physiological parameters were integrated over the leaf area of the respective fraction over the ground area for calculating the *A*_c_ and *A*_j_ on a fraction basis (Wu et al., 2018). Unlike the leaf‐level *A*_c_ and *A*_j_, the fraction‐level *A*_c_ and *A*_j_ represent the collective rates of all leaves in the fraction, having incorporated within canopy variations in intercepted light and photosynthetic parameter value through canopy depth. The sunlit and shaded fraction *A*_c_ and *A*_j_, together with *C*_i_/*C*_a_, defined the instantaneous *A* of the respective leaf fraction. This was calculated hourly over the diurnal cycle and summed to predict total diurnal canopy photosynthesis and converted to daily above‐ground biomass growth following Wu et al. (2018). The model assumes photosynthesis and stomatal conductance responds instantaneously to changing light conditions to reach steady‐state levels (Wu et al., 2018). This approach was demonstrated to be adequate for predicting field‐observed crop biomass growth over the whole crop cycle (Wu et al., 2019). Fraction‐level *A*_c_ and *A*_j_ can also be plotted to give *A*–*C*_i_ curves (e.g., Supporting Information: Figures S1 and S2).

### 2.2 Dynamic crop growth and yield simulation

During each crop growth simulation cycle, the cross‐scale model simulated temporal changes in crop phenological stage, canopy leaf area expansion, canopy light interception and transpiration, canopy photosynthesis and biomass growth, crop water use, resource (carbon, water and nitrogen) supply‐demand balance, carbohydrate and nitrogen allocation among organs, growth of grains, and effects of environmental variables (sunlight, water, temperature and nitrogen). The crop attributes interact with one another following the crop physiological network developed and validated in the APSIM platform using extensive field experiment data (e.g., Hammer et al., 2019a). Demand for carbohydrates and nitrogen is defined by potential organ (stem, leaf, grain) growth parameterized for the wheat and sorghum cultivars used in this study (Brown et al., 2014; Hammer et al., 2010; van Oosterom et al., 2010a, 2010b).

**Figure 4:**
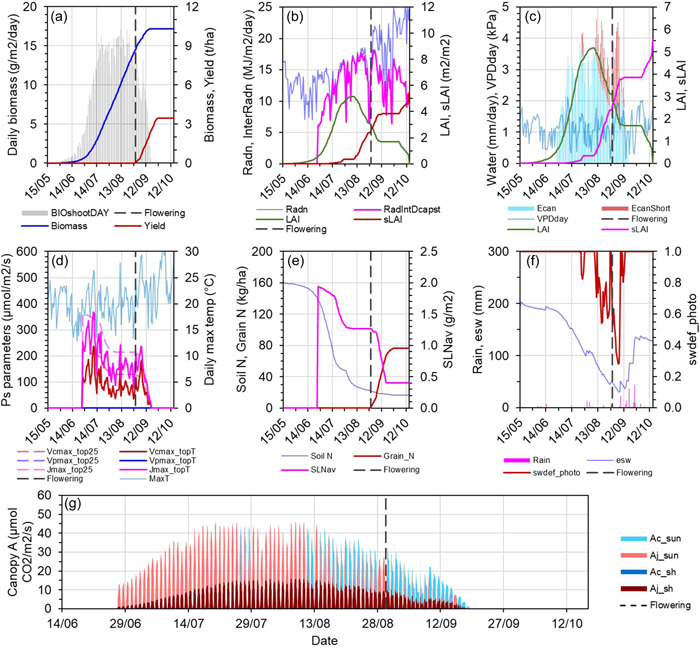
Predicted wheat crop attributes dynamics, and environmental variables over a sample crop cycle. Results are from a medium‐yielding year at the Dalby site with the medium sowing date and starting soil water (Supporting Information: Table S4). (a) Cumulative crop biomass and yield. (b) Canopy leaf area index, solar radiation, and interception. (c) Potential crop water demand is shown by the bars, which is made up of a fraction that is met by supply from soil water uptake by roots (i.e., actual water use) and a fraction that is not met (red bars). (d) Photosynthetic parameters for the uppermost leaves of the canopy at 25°C and the maximum air temperature during the day. (e) Soil N supply and crop N status including specific leaf nitrogen and N in grains. (f) Plant extractable soil water and a crop water stress factor; a value of 1 means all crop water demand is being met, while 0 means no water is available. (g) Daily canopy photosynthesis; each peak is made up of a histogram of total canopy photosynthesis on an hourly timestep over one diurnal period. An equivalent figure for sorghum is shown in Supporting Information: Figure S4. BIO_shootDAY_, daily shoot biomass growth; Radn, daily incident solar radiation, RadIntDcapst, daily intercepted radiation by the whole canopy; LAI, leaf area index; sLAI, senescenced LAI; Ecan, actual crop water use; EcanShort, fraction of the potential demand not met by supply; VPDday, indicative daytime vapour pressure deficit; Vcmax_top25, Vpmax_top25, Jmax_top25 are the values of the maximum rate of Rubisco carboxylation, maximum rate of PEP carboxylation, and maximum rate of electron transport at infinite light at 25°C; Vcmax_topT, Vpmax_topT, Jmax_topT are those photosynthetic parameter values calculated using the maximum temperature of the day (MaxT); SLNav, canopy‐average specific leaf nitrogen; esw, plant extractable soil water; swdef_photo, a crop water stress factor given by EcanFilled divided by the sum of EcanFilled and ECanShort; Ac_sun and Aj_sun, Rubisco activity and electron transport limited gross CO_2_ assimilation rate of the sunlit fraction of the canopy (only the lower of the two limitations is shown); Ac_sh and Aj_sh, the same limitations for the shaded fraction.

Supply of water to the crop is dependent on the effective rooting depth, which advances during the crop cycle, and the rate at which soil water can be extracted from the soil by the roots (Hammer et al., 2001). Water extraction occurs from multiple layers, and the total extraction is the sum of that calculated for individual layers. Potential N supply from the soil depends on the available soil N through the profile and on the extent to which roots have explored the soil, and the rate of N uptake by the roots (Hammer et al., 2010). Trajectories of simulated crop attribute through the crop cycle were extracted for detailed analysis to show whole crop photosynthesis, growth, development, and yield formation (e.g., Figure 4 and Supporting Information: Figure S4). The plots exemplify a medium‐yielding wheat and sorghum crop at the Dalby site with the median sowing date and starting soil water.

### 2.3 Multi‐environment simulation

Multiyear × location crop growth simulations, akin to extensive multi‐environment trials, were conducted using common wheat and sorghum cultivars to understand and quantify the consequences of leaf photosynthetic manipulation on crop growth and yield over a wide range of environments. This involved running simulations with representative daily weather data at selected sites across crop production regions. Australian environments were used in this study as the year‐to‐year environmental condition variability presents a diverse set of non‐stressed and stressed conditions and can generate a wide range of yield levels. The median sowing date, the median amount of stored soil water at sowing, and the most commonly used agronomy and N application for the crop were used in this multi‐environment simulation (Supporting Information: Table S4).

The weather and soil aspects of the simulations were parameterized depending on the crop in question and the production site. The target population of environments for wheat in Australia has been classified into six distinct types based on a principal component analysis of long‐term year‐to‐year production variability at the shire scale (Potgieter et al., 2002; Supporting Information: Figure S3). One production site representative of each of these six regions was selected based on its loading for the respective principal component as well as being a key centre/town for wheat production (Supporting Information: Table S4). Similar considerations were followed in selecting the four sites from north‐eastern Australia for sorghum production simulation.

Interannual weather variability at each site was represented by accessing its long‐term (1900–2020) daily weather record (including maximum and minimum air temperature, incoming solar radiation, and precipitation), which was obtained from the SILO patched point data set (http://www.longpaddock.qld.gov.au/silo/index.html; Jeffrey et al., 2001). The intention was not to simulate historical yield levels but to use historical weather data to sample interannual weather variabilities. Ambient CO_2_ was set at 400 ppm (ca. 400 μbar). Detailed parameterisations of soil characteristics (including soil depth, plant available water capacity, and typical N present in the soil at sowing) were taken from Chenu et al. (2013) and Hammer et al. (2014).

Medium‐maturing wheat (Janz) and sorghum (Hybrid MR‐Buster) cultivars were used in the multiyear × location simulation (Supporting Information: Table S4). Their physiology reflects the commonly used cultivars in Australian production environments and their physiological response to environmental variables has been well‐parameterized in APSIM crop growth models and tested (Ababaei & Chenu, 2020; Hammer et al., 2010).

Locally adapted agronomic practices for the different sites were used. Briefly, wheat is sown around May–June each year, while sorghum has a wider sowing window between October and January. Sowing dates used in this multiyear × location simulation were the median values calculated from the reported uniform distribution of dates within the sowing windows (Ababaei & Chenu, 2020; Hammer et al., 2014). A row‐planting configuration was used for both crops with sorghum having a 1‐m row spacing and 5 plants m^–2^, while wheat had 0.25‐m row spacing and a density of 100 or 150 plants m^–2^ (Supporting Information: Table S4). Starting soil water content was set to the median values, which were calculated from the frequencies reported for wheat (Chenu et al., 2013) and sorghum (Hammer et al., 2014). Soil N at the time of sowing ranged between 30 and 50 kg ha^–1^. The sorghum crop is typically fertilized with N before or at sowing with N applied to the surface soil layers, while for wheat N application can also occur later in the growing season depending on crop stage and soil water/precipitation conditions (Supporting Information: Table S4). The weather variability, crop configuration, and N application combinations present a broad spectrum of non‐stressed to stressed production conditions.

### 2.4 Modelling leaf photosynthetic manipulation

A broad list of photosynthetic manipulation strategies examined in this study covers the three key aspects of leaf photosynthesis related to Rubisco function, electron transport chain, and CO_2_ delivery. The C_3_ photosynthesis setting of the generic photosynthesis–CO_2_ diffusion model was used for most of the wheat photosynthetic manipulation simulations, except that the single‐cell CCM setting was used to model the installation of the CCM and its components. The C_4_ setting was used for all of the sorghum photosynthetic manipulations.

A description of how each manipulation strategy is theorized and modelled using the generic photosynthesis–CO_2_ diffusion module in the cross‐scale framework is set out fully in Table 1. Briefly, the manipulations include enhancing Rubisco function by enhancing its catalytic properties and/or content (manipulation outcomes 1.1, 1.2, 1.3, and 1.4; Martin‐Avila et al., 2020; Salesse‐Smith et al., 2018; Sharwood et al., 2016a, 2016b); a ‘better’ Rubisco from stacking Rubisco function enhancements (outcome 2); enhancing CO_2_ delivery by improving mesophyll conductance (outcome 3; Groszmann et al., 2017), installation of cyanobacterial bicarbonate transporters and a full cyanobacterial CO_2_ concentrating mechanism in C_3_ wheat (outcomes 4.1 and 4.2; Price et al., 2013), overexpression of PEP carboxylase in C_4_ sorghum (outcome 5), or reducing bundle sheath conductance in C_4_ sorghum (outcome 6); enhancing electron transport rate by overexpression of the Rieske FeS protein of the cytochrome *b*_6_f complex (outcome 7; Ermakova et al., 2019; Simkin et al., 2017), or extending useful photosynthetically active radiation to 700–750 nm of leaves by supplementing light‐harvesting complexes with cyanobacterial Chlorophyll *d* and *f* in all leaves of the canopy (outcome 8; Chen & Blankenship, 2011). A tangible case of stacking a selection of some of these strategies was also included (outcome 9: ‘better’ Rubisco, overexpression of Rieske FeS protein, and improved mesophyll conductance). These manipulations are modelled by specific changes in the photosynthesis–CO_2_ diffusion model parameters, which are given in Table 1 with supporting references. Manipulations have different effects on the *A*_c_ and *A*_j_. Examples of predicted consequences of these manipulations on the *A*_c_ and *A*_j_ limitations and leaf‐level *A*–*C*_i_ response are shown in Figures 2 and 3.

Leaf nitrogen costs of achieving leaf photosynthetic manipulation can be assumed neutral. Modifying Rubisco kinetic properties (outcomes 1.1, 1.2, and 1.4) and swapping chlorophyll types (outcome 8) have minimal net N cost requirements. N cost associated with increased expression of proteins for manipulation outcomes 3, 4.1, 5, 6, and 7 is likely to be small (Evans & Clarke, 2019). Increasing Rubisco content in C_4_ sorghum (outcomes 1.3 and 2) is also likely small in N cost due to a lower baseline content. Additional N cost associated with both bicarbonate transporters and whole carboxysomes (outcome 4.2) could be offset by savings from reduction in Rubisco content as detailed in Table 1 (Rae et al., 2017). Therefore, it was assumed that photosynthetic manipulations were achieved with no effects on the N demand of expanding leaf, leaf structural N requirement (or minimum leaf N), and N translocation from leaves to other plant organs (van Oosterom et al., 2010a, 2010b). However, changes in crop carbon balance due to perturbed leaf photosynthesis can impact the timing and level of nitrogen demand and allocation over the crop life cycle. Differences in the level of leaf nitrogen content will regulate the key photosynthetic parameter (Wu et al., 2019).

**Figure 5:**
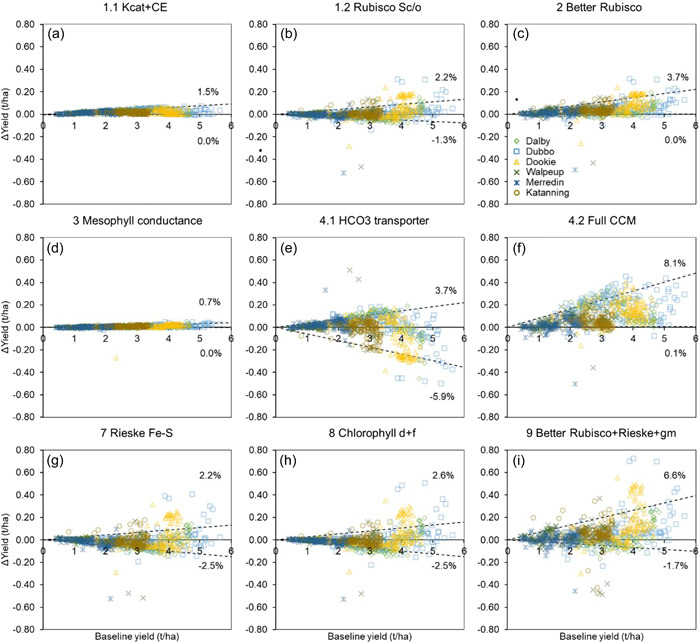
Predicted change in wheat yield (t/ha) relative to the baseline simulations for leaf photosynthetic manipulations. Panels give results for the different manipulation strategies (Table 1); results are plotted together for the six contrasting sites across the Australian wheat belt. This focused set of simulations uses representative seasonal weather data sampled from the past 120 years (1900–2020), the medium sowing date, and plant available water at sowing specific for each site (Supporting Information: Table S4). The dashed lines indicate the 10th and 90th percentile regressions for ∆yield versus baseline yield. Their slopes indicate the upper and lower percentage yield changes (*n* = 1440 crop cycles per panel; 720 baseline and 720 with manipulation).

**Figure 6:**
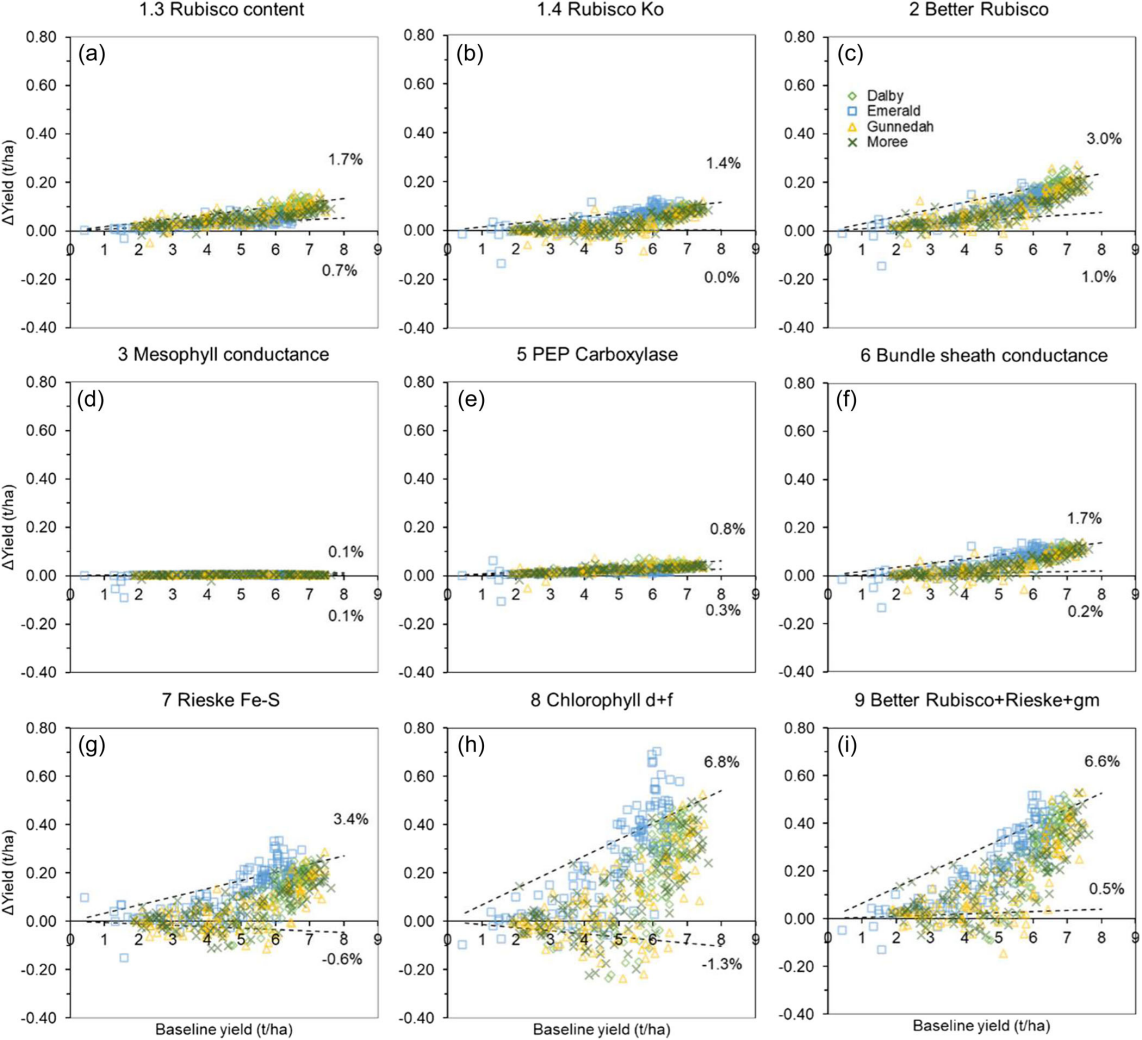
Same as for Figure 5 for predicted sorghum yield changes. Results are plotted together for the four contrasting sites across sorghum production regions (*n* = 960 crop cycles per panel).

Changes in the dynamics of crop growth and yield were predicted for the different photosynthetic manipulation strategies based on the multi‐environment simulation setup described above. Crop attribute trajectories with and without photosynthetic manipulation were generated and used in detailed analysis. Examples of these are shown in Supporting Information: Figures S5–S7. Consequences of photosynthetic manipulations for grain yield were quantified using change in simulated yield relative to the baseline parameterisation across the range of production environments in this multiyear × location simulation (Figures 5 and 6). The yield change associated with photosynthetic manipulation for each simulation crop‐year was plotted against the yield level for the baseline scenario. Quantile regression was performed in Python using the statsmodels' QuantReg class to identify the 10th and 90th percentile regressions in the plots to delineate the upper and lower percentage yield change (Figures 5 and 6).

### 2.5 Australian crop production simulation

As a case study, the consequences for national‐scale crop production of photosynthetic manipulations were quantified by using baseline production at the regional scale combined with the extent of the impact of each leaf photosynthetic manipulation strategy. Historical Australian wheat (1901–2004) and sorghum (1983–2015) production data at the regional level (Potgieter et al., 2002) were averaged and used as the baseline (Supporting Information: Table S6). For quantifying the yield impact of the manipulations, the multiyear × location simulation was expanded to include sowing dates and starting soil water levels as additional factors. Three representative levels of each of the sowing dates and starting soil water were calculated from their distributions (as described above) and used in the simulation (Supporting Information: Table S4). The overall year × sowing date × soil water × site × manipulation amounted to 194k crop cycles, for wheat and sorghum combined. This ensured a balanced representation of all possible starting conditions at sowing in the crop production simulations. The median percentage change in grain yield, and the first and third quartile values at each representative site (Supporting Information: Table S7) were predicted and applied to the corresponding regional scale production. National scale impact was calculated by weighting regional contribution to national production (Supporting Information: Table S6).

## 3 RESULTS AND DISCUSSION

Understanding and bioengineering of photosynthesis at the biochemical/leaf level have advanced significantly over the past decades with promising evidence at the leaf level of target improvements. However, assessment across the crop life cycle in multiple environments remains limited or indirect. In this study, we used a state‐of‐the‐art cross‐scale crop growth model (Wu et al., 2019) to generate a novel understanding and address a key knowledge gap in how potential photosynthetic manipulation can affect crop growth and yield dynamics in a wide range of environments. Predictions on leaf and canopy, to crop growth and yield are presented and discussed.

### 3.1 Seasonal photosynthesis, crop growth and yield dynamics explained

Crop cycle simulations quantify seasonal trajectories of wheat and sorghum crop attributes and generate an understanding of interactions between the crop and environment and how they regulate leaf/canopy photosynthesis (Figure 4 and Supporting Information: Figure S4). Figure 4 shows an example simulation result of a wheat crop grown in an Australian subtropical environment (Dalby, Australia) with summer‐dominant rainfall and typical late‐season water stress around the time of flowering and/or during grain filling (Chenu et al., 2013; Hammer et al., 2014). While every season and situation simulated generate specific effects on the dynamics of crop growth, it is instructive to first understand the interacting processes of an example season and with no photosynthetic manipulation to describe the details of a crop cycle simulation with the cross‐scale model used here.

At the beginning of the crop cycle, the cumulative crop biomass increased rapidly, followed by a near‐linear growth phase before growth slowed towards the end of the cycle (Figure 4a). Hence, while simulated grain mass increased after flowering it tended to plateau as growth declined during the grain‐filling period. Daily biomass growth was driven by canopy photosynthesis. Canopy photosynthesis over the diurnal period was calculated on an hourly timestep by summing the instantaneous gross CO_2_ assimilation rates of the sunlit and shaded fractions, integrated over the hour, and summed over the diurnal period. The CO_2_ assimilation rate of the fractions was determined by upscaling *A*_j_ and *A*_c_ from the leaf level and using the default *C*_i_ respective to the crop species. Details of the baseline leaf photosynthesis simulation can be found in the following sub‐section. Canopy photosynthesis changed dynamically over a diurnal period showing a peak mainly due to changing incoming radiation as the sun crosses the sky (Figure 4g).

Over the entire crop cycle, the magnitude of the diurnal canopy photosynthesis peaks changed dynamically due to feedback regulation from the status of the crop and the environment. The dynamics was driven by canopy LAI, SLN, and crop water status. Increasing LAI increased canopy radiation interception (Figure 4b). The LAI trajectory was determined by planting density, leaf appearance and expansion rates, and leaf size. The SLN level determined the key photosynthetic parameters (*V*_cmax25_, *J*_max25_, *V*_pmax25_, *V*_bmax25_ and *g*_m25_). SLN dynamics was a consequence of leaf area growth, crop N supply, and demand for N by competing growing organs (Figure 4e). The drop in SLN after flowering was due to translocation of N from leaves to satisfy the demands of developing grain. Exemplary values of the key photosynthetic parameters for the uppermost leaves of the canopy on a leaf area basis are shown for the standard temperature of 25°C (Figure 4d). As the photosynthetic parameters are temperature dependent, values calculated using the maximum temperature of the day are also shown (Figure 4d). The effect of daily temperature on canopy photosynthesis was less apparent than those of LAI and SLN. Silva‐Pérez et al. (2017) found leaf photosynthetic rate was relatively stable across a wide range of temperatures.

Canopy photosynthesis was impacted by crop water stress in the second half of the crop cycle as soil water was depleted in the exemplary crop cycle simulations. The potential demand for water uptake was driven by the transpiration rate required to maintain *C*_i_ and CO_2_ assimilation rate. If the transpiration demand could not be met by uptake and supply from the roots, then whole‐crop transpiration was limited (Figure 4c). This can limit stomatal conductance, operating *C*_i_, and CO_2_ assimilation rate (e.g., Supporting Information: Figure S1a). The severity of crop water limitation, which was indexed by the supply/demand ratio (swdef_photo; Figure 4f), also caused leaf senescence, which reduced radiation interception (Figure 4b). The reduction in growth rate and plateau in cumulative biomass towards maturity was due to a combination of reductions in canopy LAI, which reduced light interception; SLN, which reduced leaf and canopy photosynthetic performance; and crop water status, which reduced conductance and photosynthesis. Overall, these slowed down the grain mass/yield trajectory (Figure 4a and Supporting Information: Figure S4a).

Within a diurnal period, canopy photosynthesis was made up of contributions from the sunlit and shaded fractions. The shaded fraction was almost always *A*_j_ limited, while the sunlit fraction could be *A*_c_ or *A*_j_ limited. In the wheat example, the sunlit fraction was mostly *A*_j_ limited in the first half of the crop cycle (Figure 4g). However, when the crop was under water stress in the second half of the crop cycle, *A*_c_ limitation became dominant (Figure 4f,g). As explained earlier, this was due to reduced stomatal conductance and *C*_i_ (e.g., Supporting Information: Figure S1a). The predicted *A*_c_–*A*_j_ dynamics capture the important seasonal water stress effects on canopy photosynthesis when they occur. These *A*_*c*_ and *A*_j_ dynamics also occurred in the sorghum example (Supporting Information: Figure S4g). In addition, the switch between *A*_c_ and *A*_j_ limitation was more sensitive to temperature drops in sorghum. The brief dip in air temperature early in the season (Supporting Information: Figure S4d) caused an *A*_c_ limitation in the sunlit fraction (Supporting Information: Figure S4g). The simulated sensitivity to low temperatures is consistent with C_4_ photosynthesis temperature analysis (Kubien et al., 2003). Such complex dynamics of crop growth and yield will unfold differently with different photosynthetic manipulations and seasonal weather patterns.

### 3.2 Predicted leaf and canopy photosynthesis with manipulations

First, leaf steady‐state photosynthetic response to intercellular CO_2_ (*A–C*_i_) without manipulations (the baseline scenario) was predicted. This is shown for C_3_ wheat and C_4_ sorghum, calculated using the photosynthetic parameter values in Supporting Information: Table S5. For C_3_ wheat at a PPFD of 1800 μmol m^–2^ s^–1^ and 25°C, *A* was enzyme limited (*A*_c_) at low *C*_i_ and electron transport limited (*A*_j_) at high *C*_i_ (Figure 2). Transition from *A*_c_ to *A*_j_ occurred slightly above *C*_i_ = 300 μbar suggesting *A*_c_ limitation at ambient CO_2_ (i.e., *C*_i_ = 280 μbar using *C*_a_ = 400 μbar and *C*_i_/*C*_a_ = 0.7). For C_4_ sorghum at a PPFD of 1800 μmol m^–2^ s^–1^ and 30°C, *A*–*C*_i_ showed a steep *A*_c_‐limited initial CO_2_ response below a *C*_i_ of ~125 μbar followed by *A*_j_ limitation above that *C*_i_ (Figure 3). Thus *A* was limited by *A*_j_ at ambient CO_2_ (i.e., *C*_i_ = 160 μbar with *C*_i_/*C*_a_ of 0.4). This is consistent with evidence that electron transport can limit C_4_ photosynthesis under high‐light conditions (Ermakova et al., 2019). The simulated baseline *A*–*C*_i_ for wheat and sorghum were comparable to previously published data (Silva‐Pérez et al., 2017; Sonawane et al., 2017).

Rubisco function manipulations were predicted to predominantly affect *A*_c_ at low *C*_i_ (Figures 2a–c and 3a–c). The CO_2_ delivery‐related manipulations affected both *A*_c_ and *A*_j_ (Figures 2d–f and 3d–f). The electron transport chain‐related manipulations affected *A*_j_ at high *C*_i_ (Figures 2g–h and 3g–h). Stacking all three aspects affected both *A*_c_ and *A*_j_ (Figures 2i and 3i). Specifically, manipulation of C_3_ wheat Rubisco carboxylation rate and carboxylation efficiency to achieve those of C_4_ maize values (Table 1: outcome 1.1) was predicted to improve *A*_c_ and lower the *C*_i_ of the *A*_c_–*A*_j_ limitation transition (Figure 2a), which are consistent with previous simulation analysis (Sharwood et al., 2016b). Enhancement of wheat Rubisco specificity for CO_2_ improved both *A*_c_ and *A*_j_, but more so for the latter (Figure 2b). Manipulation of C_4_ Rubisco improved *A*_c_ (Figure 3a), comparable to observations in maize transgenics with increased Rubisco content (Salesse‐Smith et al., 2018). The combination of the enhancement of Rubisco properties (i.e., the ‘better’ Rubisco; Table 1: outcome 2) generated an additive effect of the component enhancements in both wheat and sorghum (Figures 2c and 3c).

On the CO_2_ delivery‐related manipulations, increasing mesophyll conductance (Table 1: outcome 3) had minimal impact on *A*–*C*_i_ response in both wheat and sorghum (Figures 2d and 3d). A previous simulation of photosynthetic CO_2_ assimilation rate across a wide range of mesophyll conductance values had also shown little effect on *A* unless mesophyll conductance was low (Groszmann et al., 2017). Installing the cyanobacterial bicarbonate transporters alone (Table 1: outcome 4.1) was predicted to improve *A*_c_ (Figure 2e) as the active transport mechanism elevated CO_2_ level at the site of Rubisco carboxylation. This was consistent with previous modelling results of ${\text{HCO}}_{3}^{-}$ transporter addition to C_3_ photosynthesis (Price et al., 2011). However, a reduction in *A*_j_ was predicted as the elevated CO_2_ could not compensate for the extra ATP requirement of the bicarbonate transporters (Figure 2e). The installation of the full cyanobacterial CCM (Table 1: outcome 4.2) was predicted to generate the greatest changes in the *A*–*C*_i_ response (Figure 2f). This extent of effect agreed with a previous study using a more elaborate model of a CCM (McGrath & Long, 2014). In C_4_ sorghum, the CO_2_ delivery‐related manipulations (Table 1: outcomes 5 and 6) affected *A*_c_ with smaller changes in *A*_j_ (Figure 3d–f).

Predicted C_3_ and C_4_ *A*–*C*_i_ with Rieske FeS protein overexpression (Table 1: outcome 7) increased in *A*_j_ and reflected responses observed experimentally in transgenic plants (Ermakova et al., 2019; Simkin et al., 2017) (Figures 2g and 3g). Addition of chlorophyll *d* and *f* (Table 1: outcome 8) had only a limited effect on *A*_j_ in C_3_ wheat as the electron transport rate was near saturation under the high‐light condition used (Figure 2h). A larger effect on *A*_j_ was predicted for C_4_ sorghum (Figure 3h), which is consistent with a higher light saturation point in C_4_ photosynthesis (Ermakova et al., 2019). The combination of the ‘better’ Rubisco, Rieske Fe–S protein, and mesophyll conductance (Table 1: outcome 9) was predicted to increase both *A*_c_ and *A*_j_ in both wheat and sorghum (Figures 2i and 3i).

It is important to note that demonstrating increases in CO_2_ assimilation rates using specific conditions typically used to quantify *A*–*C*_i_ response is not sufficient for understanding crop growth and yield consequences. The effect of manipulation strategies on the CO_2_ assimilation rate needs to be assessed against many factors. These include changes in the incident solar radiation due to the relative movement of the sun across the sky and air temperature across the growing season. In addition, it is the photosynthesis of the whole canopy that drives crop biomass growth, which is influenced by canopy leaf area index (LAI, m^–2^ leaf m^–2^ ground) and specific leaf N (SLN, g N m^−2^ leaf), both of which change throughout the crop life cycle. The diurnal canopy photosynthesis modelling approach used here calculates canopy photosynthesis by predicting and summing CO_2_ assimilation rates of the sunlit and shaded leaf area fractions of the canopy as described in the Methods. Exemplary sunlit‐fraction *A*–*C*_i_ is shown in Supporting Information: Figures S1 and S2. Relative to the leaf level, the sunlit‐fraction *A*–*C*_i_ has higher *A*_c_ and *A*_j_ due to integration of the enzyme‐limited and electron transport‐limited rates over its leaf area. However, *A*_c_ typically increases more relative to *A*_j_ and causes a reduction in the transition *C*_i_ (compare Supporting Information: Figures S1 and S2 with Figures 2 and 3). This occurs because incident PPFD on a leaf area basis does not scale linearly with the leaf area of the sunlit fraction due to leaf orientations in a crop canopy. Therefore, the *A*_c_–*A*_j_ transition for the sunlit fraction would shift to lower *C*_i_ (e.g., compare Figure 2a and Supporting Information: Figure S1a). The shaded‐fraction *A*–*C*_i_ would be dominated by *A*_j_ limitation due to low incident PPFD.

Photosynthetic manipulation effects on fraction‐level *A*_c_ and *A*_j_ were comparable, in relative terms, to those described for the leaf level (Supporting Information: Figures S1 and S2). The interactions between the *A*_c_, *A*_j_, operating *C*_i_, environmental conditions, and canopy status, underpin the dynamics of canopy photosynthesis, and stomatal conductance/crop water use, and these determine crop growth and resource demands over the crop cycle as discussed below. The effect of water stress is simulated by restricting stomatal conductance calculated by the Penman‐Monteith Combination equation (Wu et al., 2019), in which case the operating *C*_i_ would be reduced, thus leading to reduced *A* and possible limitation by *A*_c_ (e.g., Supporting Information: Figure S1a). Under limited transpiration and reduced stomatal conductance, *A*_c_ enhancement can still increase *A* by reducing *C*_i_ and improving intrinsic water use efficiency (e.g., Supporting Information: Figure S1a). This suggests the benefit of *A*_c_ enhancement is larger when water limitation is affecting photosynthesis. *A*_j_ enhancement is more relevant and beneficial without water limitation and when stomatal conductance can increase with enhanced CO_2_ assimilation rate (e.g., Supporting Information: Figure S1i). However, the higher stomatal conductance drives higher transpiration demand, which is a cost to crops with *A*_j_ enhancement. The changes in the steady‐state *A*–*C*_i_ response with and without manipulation described above can lead to crop growth and yield responses as discussed below.

### 3.3 Crop yield response to photosynthetic manipulation is more complex than expected

Firstly, wheat and sorghum yields without manipulations (the baseline scenario) in contrasting conditions across multiple production environments were predicted. Wheat yield from the six representative sites across Australia varied widely from 0.5 to 6 t/ha. Dalby, Dubbo, and Dookie were the higher‐yielding sites (up to 6 t/ha), Katanning was in the mid‐range (2–3.25 t/ha), and Walpeup and Merredin were the lower‐yielding sites (0.5–3.5 t/ha, but mostly below 2.5 t/ha) (Figure 5). The variations in the baseline yield across the sites were due to the local environment, agronomic management practices with N input as the major factor (Supporting Information: Table S4), and seasonal climate variability within sites (Chenu et al., 2013). The simulated baseline sorghum yield from the four representative sites in NE Australia also varied widely from 1 to 8 t/ha. However, although agronomic management practices (Supporting Information: Table S4) were similar, there was significant variation at all sites due to the extent of seasonal climate variability. The simulated wheat and sorghum yields in the different local environments were comparable to those reported previously in comprehensive crop‐environment analysis studies (Chenu et al., 2013; Hammer et al., 2014) indicating the cross‐scale model extension is robust across a spectrum of non‐stressed and stressed crop conditions, as previously demonstrated (Wu et al., 2019).

Wheat and sorghum crops with photosynthetic manipulation were predicted across a diverse range of production environments (Supporting Information: Figure S3 and Table S4). The results of the magnitude of yield change relative to the baseline scenario (∆yield) were dependent on both the manipulation target and the environment as shown in Figures 5 and 6. The top decile of ∆yield was up to an equivalent of an 8.1% yield increase with the installation of the full cyanobacterial‐type CCM (Figure 5f). The simultaneous enhancement in Rubisco functions, Rieske FeS protein, and mesophyll conductance gave both wheat and sorghum ∆yields of up to 6.6% (Figures 5i and 6i). This yield effect was also predicted for Chlorophyll *d* and *f* in sorghum (Figure 6h). The Rubisco function (Figures 5a–c and 6a–c) and electron transport chain (Figures 5g,h and 6g) targets had similar, but smaller ∆yield effects (~1.4%–3.7%) in both wheat and sorghum. Apart from reducing bundle sheath conductance in sorghum (Figure 6f), the other CO_2_ delivery‐related targets had a limited effect on wheat and sorghum ∆yield (Figures 5d and 6d,e). The comparative magnitudes of the top decile of ∆yield presented in Figures 5 and 6 agreed well with the leaf‐level enhancements predicted earlier (Figures 2 and 3). The physiological reasons for the predicted ∆yield from a whole‐crop context, and its apparent variability across different environments, are discussed below.

The physiological underpinning for the predicted positive ∆yield was a predicted increase in grain number in both wheat and sorghum. A detailed inspection of the predicted crop attribute trajectories revealed that leaf photosynthetic manipulations that enhanced *A*_j_ increased canopy CO_2_ assimilation and biomass growth, and canopy size (or LAI) early in the crop cycle (e.g., Figure 4 and Supporting Information: S5). This allowed crops to achieve higher growth rates, transpiration, and biomass around anthesis, which increased grain number (van Oosterom & Hammer, 2008). In situations with positive ∆yield, water and nitrogen were less limiting after anthesis, hence the crop could carry on photosynthesising to fill all grains so grain size was not impacted (e.g., Supporting Information: Figure S5). In these cases, enhanced leaf photosynthesis increased yield. Since the installation of the full CCM gave the largest effect on *A*_j_ (Figure 2f), it generated the largest ∆yield as anticipated (Figure 5f). A large effect on rice biomass growth with a full CCM was also predicted in a previous study (Yin & Struik, 2017). Figures 2, 3, 5, and 6 show how each of the manipulation outcomes impacted yield.

However, considerable variability in ∆yield was predicted even in high‐yielding conditions (e.g., Figure 5f, high‐yield region). The physiological underpinnings of this were associated with interactions between the altered crop growth and the timing and severity of water and/or nitrogen stress around the critical flowering–grain filling period. Despite increased canopy photosynthesis and biomass growth in the first half of the crop cycle, photosynthetic enhancement caused increased transpiration and exacerbated the severity of late‐season water stress in less water‐abundant seasons due to higher gas‐exchange rates earlier in the season (e.g., Supporting Information: Figure S6). This resulted in reduction in stomatal conductance and CO_2_ supply for photosynthesis later in the cycle. This could be further compounded by a reduction in LAI due to enhanced leaf senescence reducing canopy light interception. Greater early biomass growth increases crop N demand and generates a later dilution of leaf nitrogen causing lower SLN and photosynthesis in the second half of the crop cycle. The overall result would be lower canopy photosynthesis and crop growth rates during the grain‐filling period, resulting in reduced grain size. In some instances, such grain size reduction would offset grain number increase, thus explaining the ∆yield variability (e.g., Figures 5g,h and 6g,h). This highlights the fact that the effects of photosynthetic enhancement will be modulated by whole‐plant physiological limits and the environmental context, especially in resource (water and nitrogen) limited production environments.

The nature of ∆yield and its variability in high‐yielding conditions differed between the manipulation targets. Manipulations that enhanced *A*_c_, including the Rubisco function and the full CCM, resulted in ∆yield that ranged from near nil to small positive values (Figures 5a–c,f and 6a–c). However, some negative ∆yields were predicted with manipulations that enhanced *A*_j_, including the electron transport chain targets (Figures 5g,h and 6g,h). The manipulation target stacking scenario resulted in wider ∆yield variations than its component targets (Figures 5i and 6i). Given the consequence of the manipulation on timing and severity of water and/or nitrogen stress, Rubisco functions, the installation of the full cyanobacterial‐type CCM, or reduced bundle sheath conductance manipulations (Figures 5c,f and 6f) should also result in negative ∆yield outcomes as with the electron transport chain targets (Figures 5g,h and 6g,h). However, this was predominantly not the case due to the benefit of *A*_c_ enhancement in improving canopy photosynthesis, especially under water stress conditions. The Rubisco and CO_2_ delivery targets resulted in improved canopy photosynthesis and biomass growth during the stress period through better intrinsic water use efficiency (e.g., Figure S1a). This means enhanced canopy photosynthesis, crop growth, and less impact on grain size. As expected, the manipulation target stacking scenario slightly improved the negative ∆yield compared with enhancing the electron transport chain targets (Figures 5i and 6i).

Variability of ∆yield in the low‐yielding conditions was also dominated by the timing and severity of water and/or nitrogen stress as for the high‐yielding conditions. These were characterized by the 10th and 90th percentile regressions (Figures 5 and 6). The regressions also highlighted that manipulation strategies generating enhanced *A*_j_ were especially beneficial for the high‐yielding environments as there were instances the ∆yield increased well above the general trends (Figures 5g–i and 6g–i). This was due to *A*_j_ being the predominant limitation over the crop cycle (e.g., Figure 4g and S4g) and in seasons where more water was available, increased crop water use was less detrimental. Although there are modest gains to be made with the best photosynthetic manipulation strategies (e.g., Figures 5f and 6h,i), another key for crop improvement is better addressing the variation in ∆yield generated by plant–environment interactions.

Installation of the cyanobacterial ${\text{HCO}}_{3}^{-}$ transporters showed a distinct ∆yield pattern (Figure 5e). The positive ∆yield was not due to increased grain number as described earlier. Analysis revealed that canopy photosynthesis, biomass growth, and grain number were reduced (e.g., Supporting Information: Figure S7). Canopy photosynthesis was reduced early in the crop cycle due to the extra ATP costs of the transporters reducing the already limiting *A*_j_ (Figure 4g). The decline in canopy‐level *A*_j_ was consistent with the leaf‐level result (Figure 2e). However, reduced *A*_j_ and growth helped conserve water and nitrogen for the second half of the crop cycle. This meant better LAI retention, canopy light interception, and water availability, so growth rates were better sustained during the critical flowering–grain filling period and increased grain size, which compensated for the reduction in grain number due to reduced early seasons growth. However, the ${\text{HCO}}_{3}^{-}$ transporters installation was also the only approach that resulted in large negative ∆yield effects (Figure 5e). In contrast to the negative ∆yield with some of the other manipulation cases (e.g., Figure 5g,h), this occurred in those seasons with more plentiful water and nitrogen conditions where grain size was close to its potential so any reduction in grain number led to sinking limitation and reduced yield.

### 3.4 Case study: Potential impact on Australian crop production and globally

**Figure 7:**
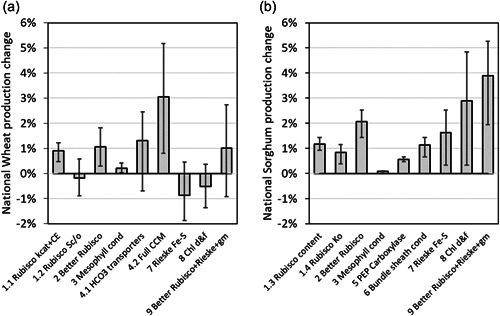
Predicted percentage change in Australia‐wide (a) wheat and (b) sorghum production associated with leaf photosynthetic manipulations (Table 1). This expanded set of simulations uses representative seasonal weather data sampled from the past 120 years, three representative levels of each sowing date, and starting soil water specific for each site (Supporting Information: Table S4). Median values are given by bars. Whiskers show the first and third quartile values, which are calculated using the corresponding quartile values from all production sites (wheat: *n* = 12,960 crop cycles per bar; sorghum: *n* = 8640 crop cycles per bar).

Quantifying the potential impact of leaf photosynthetic manipulation strategies on Australian wheat and sorghum production at a national scale revealed differences among the manipulation targets and crops. The potential magnitude of enhancement in the predicted steady‐state *A*_c_ and *A*_j_ (Figures 2 and 3) reflected expectations based on transgenic and previous modelling studies. However, the largest levels of crop production increase were modest with median increases of 3–4% at a national scale (Figure 7). The modest levels of increase, which exhibit a range of potential outcomes and instances of negative change, were associated with a more rigorous sampling of effects of diverse environmental and agronomic conditions that generate a realistic frequency of incidence of water and nitrogen limitations at the national scale. The full CCM installation (4.2) generated the largest increase in Australian wheat production with a median gain of ~3%, while some of the Rubisco (1.1 and 2) and bicarbonate transporter (4.1) manipulation strategies generated a ~1% increase. Rieske Fe–S (7) and chlorophyll *d* & *f* (8) manipulation strategies resulted in slightly reduced overall production at the national scale. The electron transport chain targets resulted in wider production change variabilities as they tended to exacerbate crop water and/or nitrogen stress. The manipulation stacking strategy (9) did not result in a further increase in the median value compared to just ‘better’ Rubisco (2), but it also increased the production variability. In sorghum, incorporating chlorophyll *d* and *f*, and the manipulation stacking strategy generated the largest production gain (3%–4%). This contrasted with the wheat predictions as nitrogen limitation was less detrimental in sorghum production. Nitrogen deficiency was also found to reduce yield gains with enhanced photosynthesis from elevated CO_2_ in a large number of C_3_ crops (Ainsworth & Long, 2021). Other manipulation targets such as those related to Rubisco (1.3, 1.4, and 2), bundle sheath conductance (6), and Rieske Fe–S (7) will likely result in ~1%–2% increase in Australian sorghum production. In both crops, the likely impact of manipulating mesophyll conductance (3) was consistently low.

Benchmarking impacts of photosynthetic enhancement against current year‐on‐year crop yield advances provide a useful context for crop breeding efforts. The historical annual rates of increase in the national average yield of Australian wheat and sorghum are 1.2% and 2.1%, respectively (Potgieter et al., 2016). These rates quantify the extent of continual technological advances arising from crop improvement due to empirical breeding based on selection for yield, advances in agronomy such as stubble management practices to enhance soil water availability, and some environmental trend effects (e.g., rising CO_2_). Hence, implementing the best of the photosynthetic manipulation targets will likely result in an equivalent of 2.5 and 2 years of conventional production gains for Australian wheat and sorghum, respectively.

An effective first‐order approach in predicting yield impacts across international locations is by applying the predicted Australian yield changes to correlated international environments globally. Australian production environments present a broad spectrum of non‐stressed to stressed production conditions. Marginal Australian environments like southern and western Australia are correlated with South America, southern Africa, Iran, and high‐latitude European and Canadian locations (Mathews et al., 2007). For these locations, the installation of full CCM is likely to be the most beneficial, generating between 0.1% and up to 8.1% gains based on the top and bottom 10th percentile regression (Figure 5f). High‐yielding environments such as eastern Australia are correlated with international locations including the Indo‐Gangetic plains, West Asia, North Africa, Mexico, and locations in Europe and Canada (Mathews et al., 2007). In these environments and if water and nitrogen were also abundant, the manipulation stacking strategy is likely to be the most beneficial, generating up to 15% gains in wheat yield (Supporting Information: Figure S8). This is also evident in Figure 5i showing instances of large ∆yield well above the top 10th percentile regression. Understanding and quantifying production environment context dependencies is important for maximising yield improvement.

### 3.5 Cross‐scale analysis helps understand and quantify the effects on crop yield

This study used a state‐of‐the‐art cross‐scale model to predict the effects of a broad list of photosynthetic manipulation strategies on seasonal crop growth and yield dynamics and quantified the potential impact (or lack of it) on crop yield across a broad spectrum of non‐stressed to stressed production conditions. Based on the potential magnitude of enhancement in the steady‐state leaf photosynthetic rates, predicted yield increases are likely modest even in the top decile of seasonal outcomes, ranging between 0% and 8% depending on the crop type and manipulation. Importantly, effects on yield can vary non‐intuitively from the top seasonal outcomes to nil or losses depending on the availability of water and nitrogen. Our analysis of the manipulation of the steady‐state enzyme‐ (*A*_c_) and electron transport‐limited photosynthetic (*A*_j_) rates suggests strategies that enhance both will be needed for achieving the larger of the predicted yield gains, which will likely be achieved by stacking Rubisco function and electron transport chain enhancements or installing a full CO_2_ concentrating system. Strategies that target *A*_c_ alone will likely be less impactful for increasing yield and targeting *A*_j_ alone will likely result in yield penalty in less favourable environments. The modest results and environmental context dependencies challenge common perceptions, which have been based on limited field experiments and modelling, of the magnitude of benefits likely to arise from photosynthetic manipulation.

The current cross‐scale model has allowed the assessment of the multitude of photosynthetic manipulation strategies that influence steady‐state leaf photosynthetic rates. This is conducted for recent photosynthetic manipulation research related to Rubisco function, leaf internal CO_2_ delivery, electron transport, and manipulation stacking (Table 1). Simulation of targets related to non‐steady‐state photosynthetic rates and stomatal conductance response (Lawson & Vialet‐Chabrand, 2019; Zhu et al., 2020) can be assessed following a similar analysis presented here. However, that will require further development of the cross‐scale model capability by including the ability to simulate the photosynthetic response to change in environmental factors (e.g., light level) at small time scales (e.g., Wang et al., 2021), and a canopy model that can simulate environmental fluctuations due to movement of the sun, cloud cover, and mutual shading of leaves on each specific facet of the foliar area in the canopy. Some of the manipulations can potentially be achieved in the near future (Long et al., 2015), thus the analysis here using current climate conditions is applicable. However, understanding and quantifying photosynthetic manipulation effects in future climates will inevitably be needed. For this, the cross‐scale model will need to be combined with reliable climate projection models (e.g., Hammer et al., 2020). This will also require an increased understanding of photosynthetic and stomatal response in photosynthetically engineered target crops in different CO_2_, temperature, and vapour pressure deficit conditions to generate new leaf‐ and whole‐crop level information for cross‐scale model training and validation.

This cross‐scale modelling study sets out an analysis procedure for understanding and quantifying nonintuitive interactions across biological scales of organisation from leaf photosynthesis and transpiration to crop growth, development, and yield formation in realistic production environments. We have gained new knowledge of the likely cross‐scale interaction between the growth and yield formation of the photosynthetically enhanced crops with the environment, conducted a comprehensive impact assessment on Australian wheat and sorghum production and suggested impact on wheat yield across international locations. Direct simulation beyond the Australian environments would require knowledge of the local cultivars used, long‐term weather records, soil characterisation, and agronomic management practices to the extent of the data composed in this study. Our unique cross‐scale modelling analysis sets out a testable framework across scales of biological organisation from leaf photosynthetic CO_2_ response, to crop growth and development trajectories, to grain yield, and unpacks the effects of leaf photosynthetic perturbation on crop yield outcomes. This study has improved the understanding and quantification of the potential impact of photosynthesis traits (or lack of it) for crop improvement research.

## Supporting information

Supporting information.

## Data Availability

The data that support the findings of this study are available from the corresponding author upon reasonable request.
