# Supplementary material for: A cross‐scale analysis to understand and quantify the effects of photosynthetic enhancement on crop growth and yield across environments
Source: Plant Cell Environ. 2022 Oct 20;46(1):23–44. doi: 10.1111/pce.14453 (PMC10091820; doi:10.1111/pce.14453)
Supplement: Supplementary file 1 — Supporting information. [file PCE-46-23-s001.pdf]

A cross-scale analysis to understand and quantify effects of photosynthetic enhancement on crop growth and yield across environments

Alex Wu[1]*, Jason Brider[1], Florian A. Busch[2,3,4], Min Chen[5], Karine Chenu[1], Victoria C. Clarke[2], Brian Collins[6], Maria Ermakova[2], John R. Evans[2], Graham D. Farquhar[2], Britta Forster[2], Robert T. Furbank[2], Michael Gorszmann[2], Miguel A. Hernandez[5], Benedict M. Long[2], Greg Mclean[1], Andries Potgieter[1], G. Dean Price[2], Robert E. Sharwood[7], Michael Stower[1], Erik van Oosterom[1], Susanne von Caemmerer[2], Spencer M. Whitney[2], Graeme L. Hammer[1]

*corresponding: c.wu1@uq.edu.au

[1]ARC Centre of Excellence for Translational Photosynthesis, Centre for Crop Science, Queensland Alliance for Agriculture and Food Innovation, The University of Queensland, Brisbane, Queensland, Australia

[2]ARC Centre of Excellence for Translational Photosynthesis, Division of Plant Science, Research School of Biology, The Australian National University, Acton, Australian Capital Territory, 2601, Australia

[3]School of Biosciences, University of Birmingham, Birmingham, B15 2TT, UK

[4]Birmingham Institute of Forest Research, University of Birmingham, Birmingham, B15 2TT, UK

[5]ARC Centre of Excellence for Translational Photosynthesis, School of Life and Environmental Science, Faculty of Science, University of Sydney, Sydney NSW2006, Australia

[6]College of Science and Engineering, James Cook University, Townsville, QLD 4811

[7]Hawkesbury Institute for the Environment, Western Sydney University, Richmond, NSW, 2154, Australia

Appendix A: Expanded photosynthesis–CO_2 diffusion model equations

Three photosynthetic pathways are modelled: C_3, C_4, and single-cell CCM. Equations for calculating the Rubisco-limited (A_c) and electron-transport-limited ($\underline{A_j}$) rates of CO_2 assimilation, and the chloroplastic CO_2 supply function:

The C_3 model (Farquhar *et al.*, 1980):

$$A_c = \frac{(C_c - \Gamma_*)V_{cmax}}{C_c + K_c(1 + O_c/K_o)} - R_d \qquad \text{Eqn 1}$$

$$A_j = \frac{(C_c - \Gamma_*)J}{4C_c + 8\Gamma_*} - R_d \qquad \text{Eqn 2}$$

$$C_c = C_i/C_a \times C_a - \frac{A}{g_m} \qquad \text{Eqn 3}$$

where V_{cmax} is the maximum Rubisco activity; K_c and K_o are the Rubisco Michaelis-Menten constants for CO_2 and O_2 respectively; C_a and C_i are the ambient and intercellular CO_2 partial pressures, and C_c and O_c are the chloroplastic CO_2 and O_2 partial pressures; Γ_* is the CO_2 compensation partial pressure in the absence of mitochondrial respiration (R_d) as calculated by half O_c multiplied by the reciprocal of the relative specificity of Rubisco (i.e. $0.5O_c/S_{c/o}$); J is potential electron transport rate and is related to the incident irradiance as in Eqn 4; g_m is the mesophyll conductance to CO_2. The CO_2 supply function uses a C_i/C_a ratio and the Fick's first law of diffusion.

The relationship between J and irradiance (I) is modelled by the solution to a non-rectangular hyperbolic-type function (Farquhar & Wong, 1984, von Caemmerer, 2000):

$$J = \frac{\alpha_{PSII}I + J_{max} - \sqrt{(\alpha_{PSII}I + J_{nax})^2 - 4\theta\alpha_{PSII}IJ_{max}}}{2\theta} \qquad \text{Eqn 4}$$

Where J_{max} is maximum electron transport and θ is an empirical curvature factor of the *J-I* response. α_{PSII} is the fraction of I absorbed and used by Photosystem II to drive electron transport. α_{PSII} is calculated by assuming that leaves absorb 85% of the incident I, of which 15% is not useful due to spectral quality, of which half goes to Photosystem II (i.e. 0.85 * (1 – 0.85) * 0.5 = 0.36) (von Caemmerer, 2000). Compared with the widely used relationship between J and the quantum yield of Photosystem II from chlorophyll fluorescence measurement (Maxwell & Johnson, 2000), Eqn 4 is used to describe *J–I* over a range of irradiance here.

The C_4 model (von Caemmerer, 2000):

$$A_c = \frac{(C_s - \gamma_* O_s)V_{cmax}}{C_s + K_c(1 + O_s/K_o)} - R_d \quad \text{Eqn 5}$$

$$A_j = \frac{(C_s - \gamma_* O_s)(1-x)J_t}{3C_c + 7\gamma_* O_s} - R_d \quad \text{Eqn 6}$$

$$C_s = C_i/C_a \times C_a - \frac{A}{g_m} + \frac{P - A - R_m}{g_{bs}} \quad \text{Eqn 7}$$

where C_s and O_s are the bundle-sheath chloroplastic CO_2 and O_2 partial pressures; γ_* is the reciprocal of the relative specificity of Rubisco; here J_t is to indicate that electron transport is modelled as a whole and allocated the C_3 and C_4 cycles; x is the fraction of the generated ATP required in the mesophyll; R_m is the respiration occurring in the mesophyll; g_{bs} is the bundle-sheath conductance to CO_2. This CO_2 supply function for C_4 photosynthesis is based on the C_3 version with an added active transport of CO_2 into the relative air-tight bundle sheath involving initially fixing of CO_2 by PEP carboxylase in the mesophyll. Due to the air-tight nature, evolved of O_2 would elevate O_s, which is given by Eqn 9. P is the CO_2 supply from the mesophyll that depends on catalytic properties of PEP carboxylase and the limitation state of CO_2 assimilation (Eqn 8).

PEP carboxylation rate is defined, using equations from von Caemmerer (2000), by:

$$P = \begin{cases} \min\left(\frac{C_m V_{pmax}}{C_m + K_p}, V_{pr}\right) & \text{, for } A_c \\ xJ/\varphi & \text{, for } A_j \end{cases} \quad \text{Eqn 8}$$

Where C_m is the mesophyll CO_2 partial pressure; V_{pmax} is the maximum PEP carboxylase activity and K_p is the PEP carboxylase Michaelis-Menten constants for CO_2; φ is the 2 ATP required by the C_4 cycle.

Because of the relative air-tight nature of the bundle sheath, O_2 evolved in the cell will increase its partial pressure beyond the mesophyll O_2 partial pressure (O_m). This is calculated by Farquhar (1983) and von Caemmerer (2000):

$$O_s = O_m + \frac{\alpha A}{0.047 g_s} \quad \text{Eqn 9}$$

Where the second term gives the extra O_2 proportional to A and the fraction of O_2 evolution occurring in the bundle sheath (α).

The single-cell CCM model is derived from the C_4 and C_3 models as described by Price *et al.* (2011) and von Caemmerer (2021):

$$A_c = \frac{(C_x - \gamma_* O_x) V_{cmax}}{C_x + K_c(1 + O_x/K_o)} - R_d \qquad \text{Eqn 10}$$

$$A_j = \frac{(C_x - \gamma_* O_x) z(1-x) J}{3C_x + 7\gamma_* O_x} - R_d \qquad \text{Eqn 11}$$

$$C_x = C_i/C_a \times C_a - \frac{A}{g_w} + \frac{P - V_c}{g_x} \qquad \text{Eqn 12}$$

where C_x and O_x are the carboxysomal CO_2 and O_2 partial pressures. O_x is calculated using Eqn 9. Here the ATP-limited version of A_j is used and x is the fraction of the produced ATP required by bicarbonate pumps involved in the active transport of inorganic carbon from the mesophyll cytosol into the chloroplast. This extra ATP requirement is met by additional cyclic electron flow in the electron transport chain (Yin & Struik, 2012). The overall ATP production is calculated based on scaling the yield from linear electron flow with a factor (z) that captures the additional contribution from cyclic electron flow and proton requirement per ATP generated (Eqn 13). The CO_2 supply term into the chloroplast differs from the standard formulation of the C_4 model, assuming that photorespiratory CO_2 loss occurs in the mesophyll cytosol (Price *et al.*, 2011). P is the CO_2 supply from the mesophyll cytosol that depends on catalytic properties of the bicarbonate transporters and the limitation state of CO_2 assimilation (Eqn 14) and V_c is the Rubisco carboxylation rate (Eqn 15); g_w and g_x are the mesophyll cell wall/plasmalemma and the combined carboxysome–chloroplast envelope conductance to CO_2 respectively.

The rate of ATP production with additional contribution from cyclic electron flow is given by von Caemmerer (2021):

$$J_{ATP} = \frac{3 - f_{cyc}}{h(1 - f_{cyc})} J = zJ \qquad \text{Eqn 13}$$

where f_{cyc} is the fraction of the electron flow out of Photosystem I that procedes via cyclic electron flow; h is the number of protons required per ATP generated, which is taken to be 4.

The rate of bicarbonate transporters is given by Price *et al.* (2011):

$$P = \begin{cases} \frac{C_{cyt} V_{bmax}}{C'_{cyt} + K_b} & \text{, for } A_c \\ zxJt/\varphi & \text{, for } A_j \end{cases} \qquad \text{Eqn 14}$$

Where C_{cyt} is the mesophyll cytosol CO_2 partial pressure; V_{bmax} is the maximum bicarbonate transporter activity and K_p is the bicarbonate transporter Michaelis-Menten constants for CO_2.

φ is the ATP requirement of the two types of cyanobacterial bicarbonate transporter (i.e. BicA and SbtA; requiring 0.25 and 0.5 ATP, respectively, per bicarbonate transported) (Price *et al.*, 2011).

The Rubisco carboxylation rate (V_c) is given by either the RuBP-saturated carboxylation rate or the possible rate supported by the electron transport:

$$V_c = \begin{cases} \dfrac{C_x' V_{cmax}}{C_x' + K_c(1 + O_x'/K_o)} & \text{, for } A_c \\ \dfrac{C_x' z(1-x)J}{3C_x' + 7\gamma_* O_x'} & \text{, for } A_j \end{cases} \qquad \text{Eqn 15}$$

The expanded equation of the three photosynthesis models can be generalised into a single equation by combining Eqns 1–15:

$$A_\xi = \frac{\left\{\left((p - qA_\xi) + \dfrac{\left[\left((p - qA_\xi)x_4 + x_5\right) - x_6 A_\xi - R_m - x_7\right]x_8}{s}\right) - (x_{10}A_\xi + x_{11})\right\}x_1}{\left((p - qA_\xi) + \dfrac{\left[\left((p - qA_\xi)x_4 + x_5\right) - x_6 A_\xi - R_m - x_7\right]x_8}{s}\right) + x_{12}A_\xi + x_{13} + x_3} - R_d \qquad \text{Eqn 16}$$

Where CO_2 diffusion variables are given in Table S1. The x's are lumped coefficients given in Table S2.

Table S1. Variables associated with gas (CO_2 and O_2) diffusion used in Eqn 16.

Stomatal mode	p	q	b	s
Non-limiting water: constant C_i/C_a	$C_i/C_a \times C_a$	$\dfrac{1}{g_m}$	$\dfrac{\alpha}{0.047}$	g_{bs} or g_x
Water limited: conductance from water supply-demand balance	$C_a - \dfrac{TC_a}{g_t + T/2}$	$\dfrac{1}{g_t + T/2} + \dfrac{1}{g_m}$	$\dfrac{\alpha}{0.047}$	g_{bs} or g_x

Table S2. Lumped coefficients used in Eqn 16 for the different photosynthesis models. Electron transport associated parameters (i.e. x, z and φ) are defined in Table S3.

	x_1	x_3	x_4	x_5	x_6	x_7	x_8	x_{10}	x_{11}	x_{12}	x_{13}
	C$_3$ model										
A_c	V_{cmax}	K_c	0	0	0	0	0	0	$\gamma_* O_m$	0	$\frac{K_c}{K_o} O_m$
A_j	$J/4$	0	0	0	0	0	0	0	$\gamma_* O_m$	0	$2\gamma_* O_m$
	C$_4$ model										
A_{c1} (PEP-saturated rate)	V_{cmax}	K_c	$V_{pmax}/\left(C'_m + K_p\right)$	0	1	0	1	$\frac{\gamma_* \alpha}{0.047 g_{bs}}$	$\gamma_* O_m$	$\frac{K_c}{K_o} \cdot \frac{\alpha}{0.047 g_{bs}}$	$\frac{K_c}{K_o} O_m$
A_{c2} (PEP-regeneration-limited rate)	V_{cmax}	K_c	0	V_{pr}	1	0	1	$\frac{\gamma_* \alpha}{0.047 g_{bs}}$	$\gamma_* O_m$	$\frac{K_c}{K_o} \cdot \frac{\alpha}{0.047 g_{bs}}$	$\frac{K_c}{K_o} O_m$
A_j	$(1-x)J_t/3$	0	0	xJ_t/φ	1	0	1	$\frac{\gamma_* \alpha}{0.047 g_{bs}}$	$\gamma_* O_m$	$\frac{7}{3} \cdot \frac{\gamma_* \alpha}{0.047 g_{bs}}$	$\frac{7}{3} \gamma_* O_m$
	Single-cell CCM model										
A_c	V_{cmax}	K_c	$V_{pmax}/\left(C'_m + K_p\right)$	0	0	$\frac{C'_x V_{cmax}}{C'_x + K_c(1 + O'_x/K_o)}$	1	$\frac{\gamma_* \alpha}{0.047 g_x}$	$\gamma_* O_m$	$\frac{K_c}{K_o} \cdot \frac{\alpha}{0.047 g_x}$	$\frac{K_c}{K_o} O_m$
A_j	$z(1-x)J_t/3$	0	0	zxJ_t/φ	0	$\frac{C'_c z(1-x)J}{3C_x{}' + 7\gamma_* O'_x}$	1	$\frac{\gamma_* \alpha}{0.047 g_x}$	$\gamma_* O_m$	$\frac{7}{3} \cdot \frac{\gamma_* \alpha}{0.047 g_x}$	$\frac{7}{3} \gamma_* O_m$

Table S3. Parameter associated with electron transport as in Table S2.

Model	φ	f_{cyc}	x	z
C$_4$	2	0.25φ	$\frac{\varphi}{3+\varphi}$	$\frac{3 - f_{cyc}}{4(1 - f_{cyc})}$
Single-cell CCM	0.75			

Eqn 16 can be rearranged into a quadratic equation and A_ξ can be solved to give the following general solution for the Rubisco-limited (A_c) and electron-transport-limited (A_j) rates of CO_2 assimilation for the C_3, C_4, and single-cell CCM pathways:

$$A_\xi = \frac{-\sqrt{a^2-4b}+a}{2d} \qquad \text{Eqn 17}$$

where

$$a = p\,s \;-\; q\,R_{\mathrm{d}}\,s \;+\; q\,s\,x_1 \;+\; s\,x_1\,x_{10} \;+\; R_{\mathrm{d}}\,s\,x_{12} \;+\; s\,x_{13} \;+\; s\,x_3 \;-\; R_{\mathrm{m}}\,x_8 \;+\; p\,x_4\,x_8 \;-\; q\,R_{\mathrm{d}}\,x_4\,x_8 \;+\; q\,x_1\,x_4\,x_8 \;+\; x_5\,x_8 \;-\; R_{\mathrm{d}}\,x_6\,x_8 \;+\; x_1\,x_6\,x_8 \;-\; x_7\,x_8 \qquad \text{Eqn 18}$$

$$b = (-q\,s \;-\; q\,x_4\,x_8 \;+\; s\,x_{12} \;-\; x_6\,x_8)(-R_{\mathrm{m}}\,R_d\,x_8 \;+\; R_{\mathrm{m}}\,x_1\,x_8 \;+\; p\,R_{\mathrm{d}}\,s \;+\; p\,R_{\mathrm{d}}\,x_4\,x_8 \;-\; p\,s\,x_1 \;-\; p\,x_1\,x_4\,x_8 \;+\; R_{\mathrm{d}}\,s\,x_{13} \;+\; R_{\mathrm{d}}\,s\,x_3 \;+\; R_{\mathrm{d}}\,x_5\,x_8 \;-\; R_{\mathrm{d}}\,x_7\,x_8 \;+\; s\,x_1\,x_{11} \;-\; x_1\,x_5\,x_8 \;+\; x_1\,x_7\,x_8) \qquad \text{Eqn 19}$$

$$d = q\,s - \;s\,x_{12} \;+\; q\,x_4\,x_8 \;+\; x_6\,x_8 \qquad \text{Eqn 20}$$

Appendix B: Supporting Figures and Tables

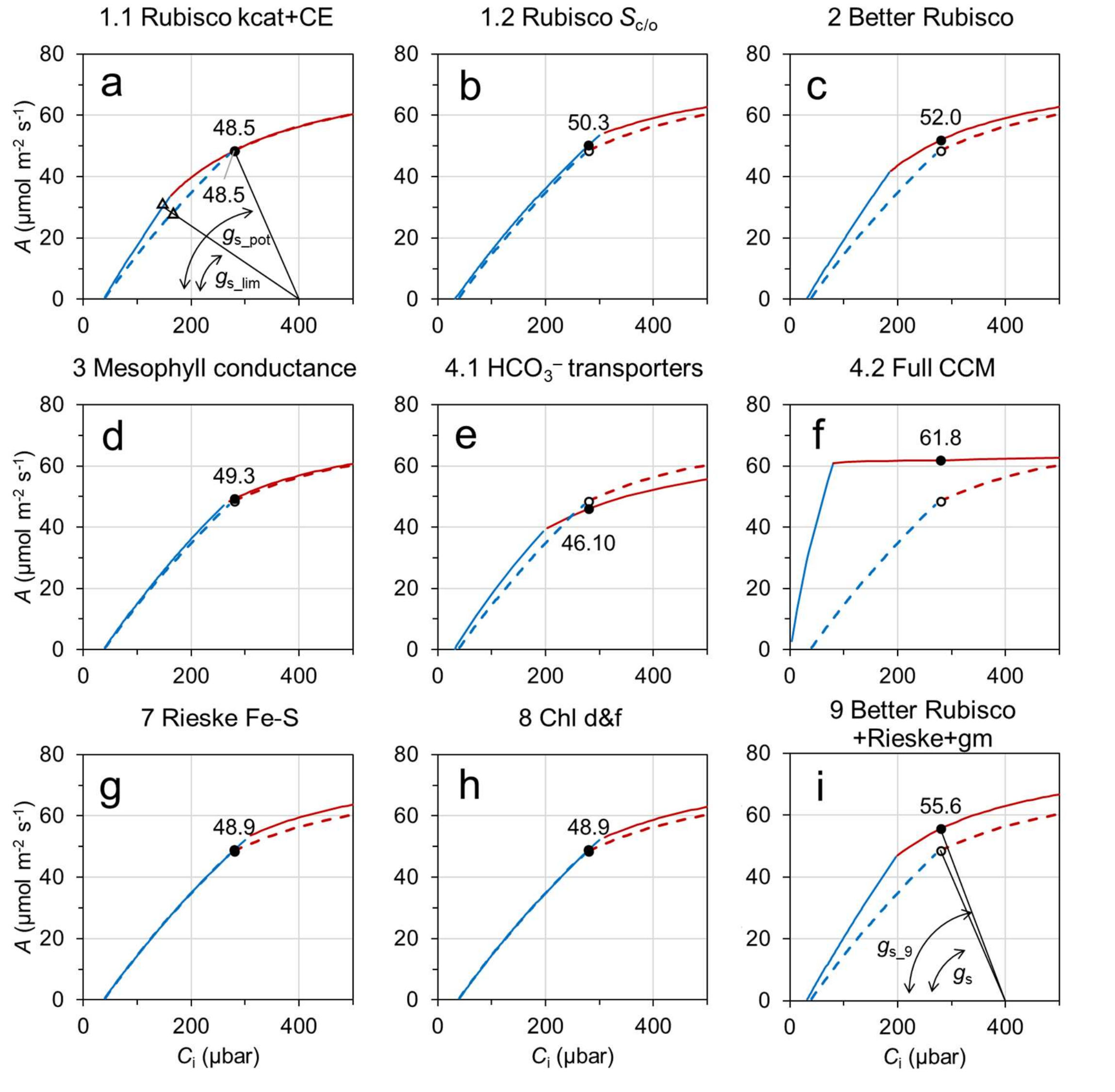

Figure S1. Simulated C_3 wheat sunlit fraction A–C_i with and without photosynthetic manipulations. The manipulations are detailed in Table 1 of the main text. Calculation of the A_c and A_j curves, on a ground area basis is described in the main text. Slopes relating air CO_2, C_i, A and g_s can be drawn: e.g. (a) shows A and g_s in non-water limited (g_{s_pot}), using a C_i/C_a of 0.7, and a water limited (g_{s_lim}) cases. Without affecting g_s, A_c enhancement increases A, but further reduces C_i/C_a under water limitation; (i) shows increase in g_s with enhancement in A_j, using a C_i/C_a of 0.7. Similarly, changes in C_i, A and g_s can be determined in other panels with and without water limitation. Results for the shaded fraction are dominated by the A_j limitation across a wide range of C_i due to low light levels (data not shown).

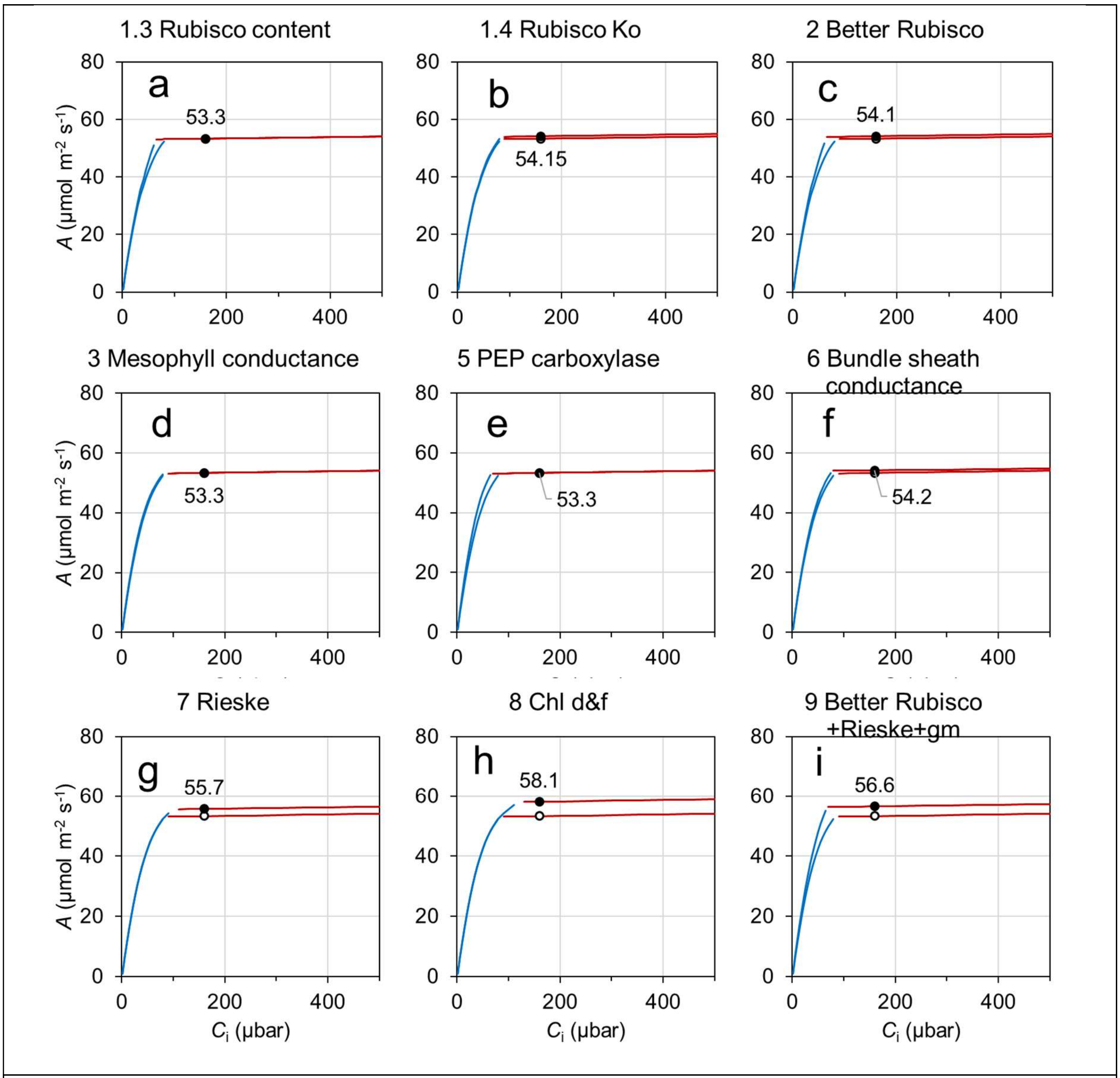

Figure S2. Simulated C_4 sorghum sunlit leaf fraction A–C_i with and without photosynthetic manipulations. The manipulations and A_c, A_j calculations are detailed in the main text. A C_i/C_a of 0.4 is used for sorghum. Figure descriptions are analogous to that of Figure S1.

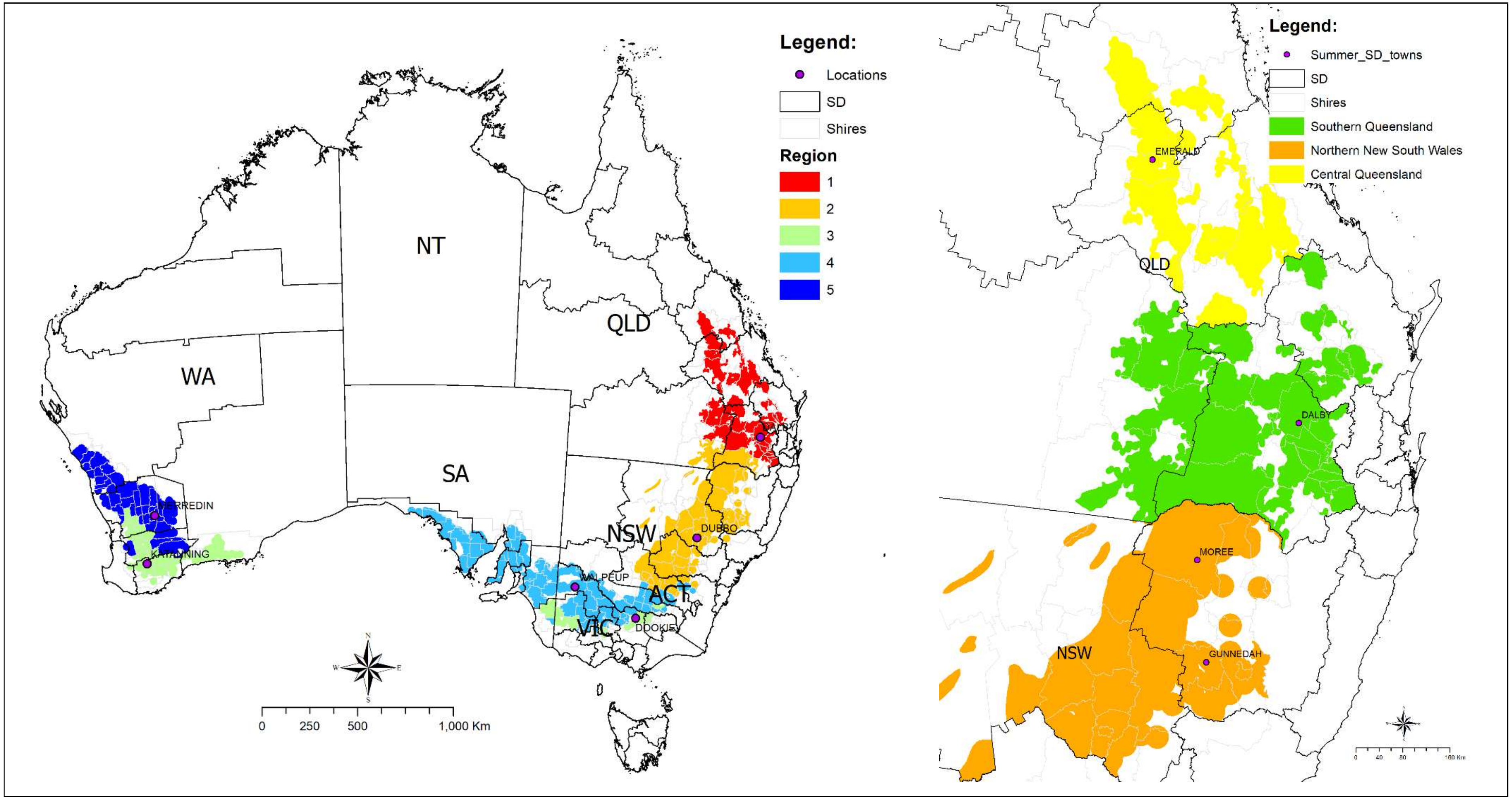

Figure S3. Australian wheat (left) and sorghum (right) production regions and the key site(s) in each region (Table S4). Statistical division (SD) boundaries are depicted in solid black lines (www.abs.gov.au).

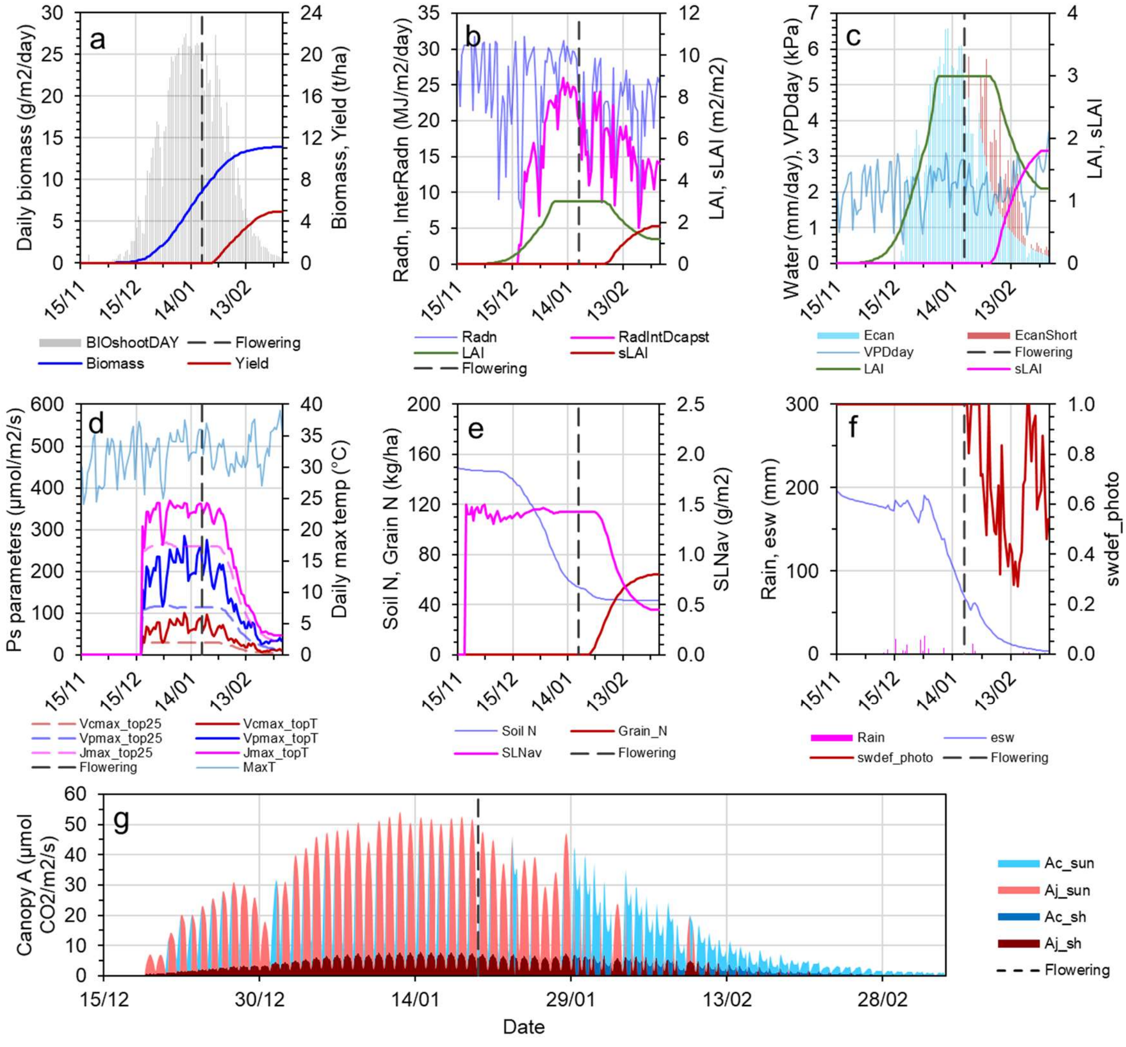

Figure S4. Predicted sorghum crop attributes dynamics, and environmental variables over a sample crop cycle. Results are from a medium-yielding year at the Dalby site with the medium sowing date and starting soil water (Table S4). (a) Cumulative crop biomass and yield. (b) Canopy leaf area index, solar radiation and interception. (c) Potential crop water demand is shown by the bars, which is made up of a fraction that is met by supply from soil water uptake by roots (i.e. actual water use) and a fraction that is not met (red bars). (d) Photosynthetic parameters for the uppermost leaves of the canopy at 25°C and the maximum air temperature during the day. (e) Soil N supply and crop N status including specific leaf nitrogen and N in grains. (f) Plant extractable soil water and a crop water stress factor; a value of 1 means all crop water demand is being met, while 0 means no water is available. (g) Daily canopy photosynthesis; each peak is made up of a histogram of total canopy photosynthesis on an hourly timestep over one diurnal period. Abbreviations: $BIO_{shootDAY}$, daily shoot biomass growth; Radn, daily incident solar radiation, RadIntDcapst, daily intercepted radiation by the whole canopy; LAI, leaf area index; sLAI, senescenced LAI;

Ecan, actual crop water use; EcanShort, fraction of the potential demand not met by supply; VPDday, indicative daytime vapour pressure deficit; Vcmax_top25, Vpmax_top25, Jmax_top25 are the values of the maximum rate of Rubisco carboxylation, maximum rate of PEP carboxylation, and maximum rate of electron transport at infinite light at 25°C; Vcmax_topT, Vpmax_topT, Jmax_topT are those photosynthetic parameter values calculated using the maximum temperature of the day (MaxT); SLNav, canopy-average specific leaf nitrogen; esw, plant extractable soil water; swdef_photo, a crop water stress factor given by EcanFilled divided by the sum of EcanFilled and ECanShort; Ac_sun and Aj_sun, Rubisco activity and electron transport limited gross CO_2 assimilation rate of the sunlit leaf fraction of the canopy (only the lower of the two limitations is shown); Ac_sh and Aj_sh, the same limitations for the shaded leaf fraction.

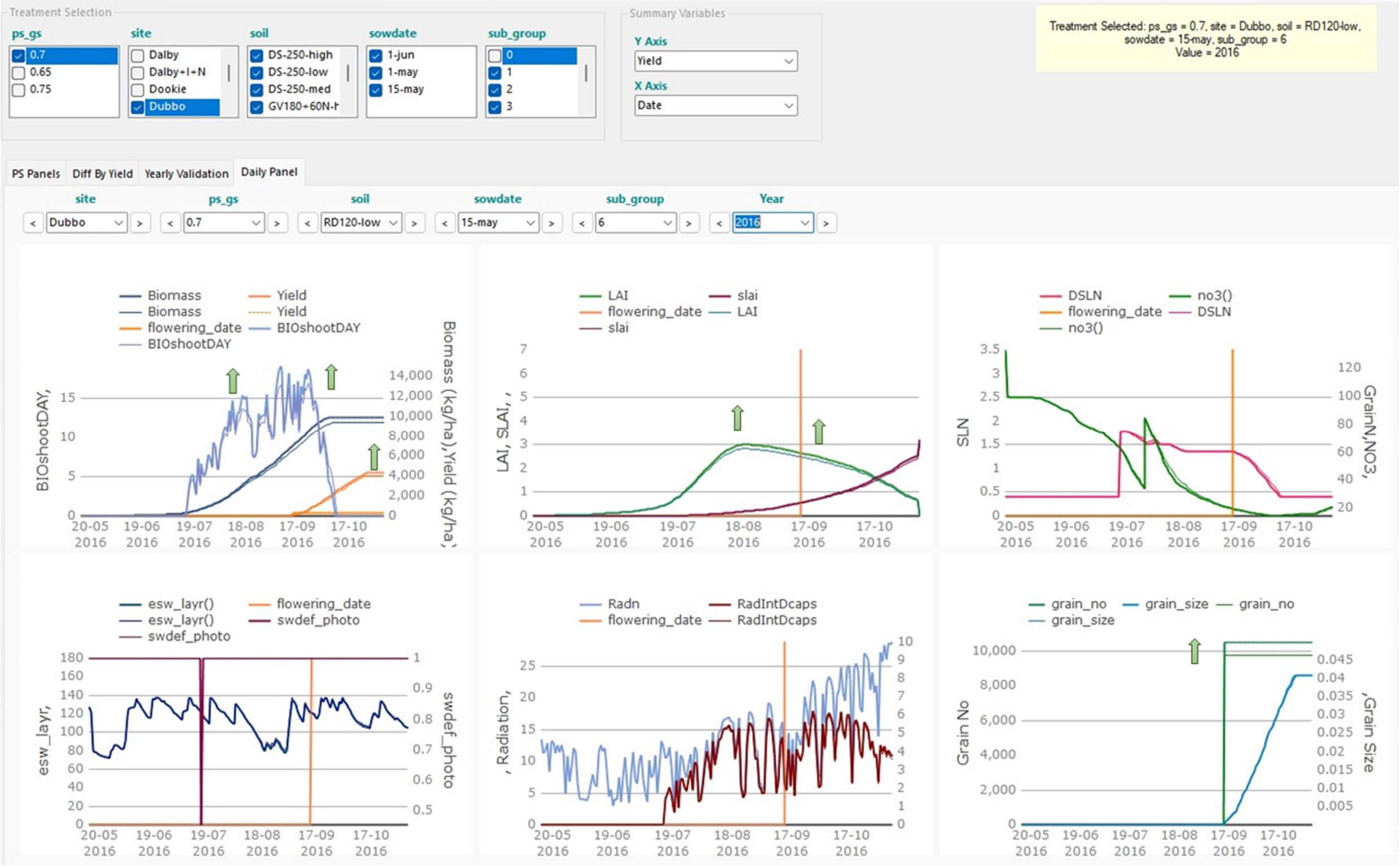

Figure S5. Wheat crop attributes trajectories with abundant water and N. A season selected from the top right-hand corner of the points in Figure 5f of the main text. Baseline trajectories are thin lines; full CCM installation (target 4.2) are thick lines. Arrows highlight changes in trajectories.

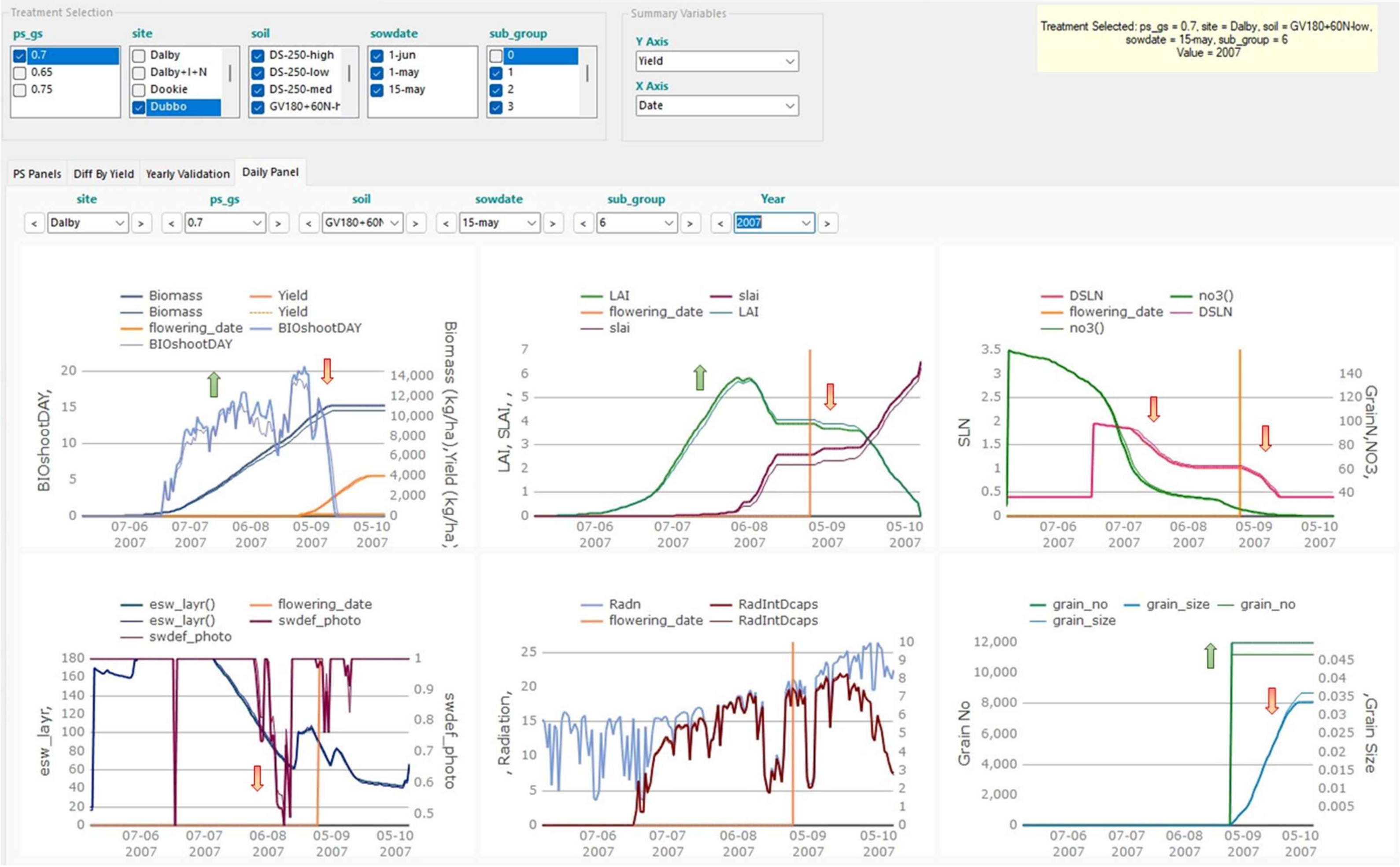

Figure S6. Wheat crop attributes trajectories with exacerbated water stress. A season selected from the bottom right-hand corner of the points in Figure 5f of the main text. Baseline trajectories are thin lines; full CCM installation (target 4.2) are thick lines. Arrows highlight changes in trajectories.

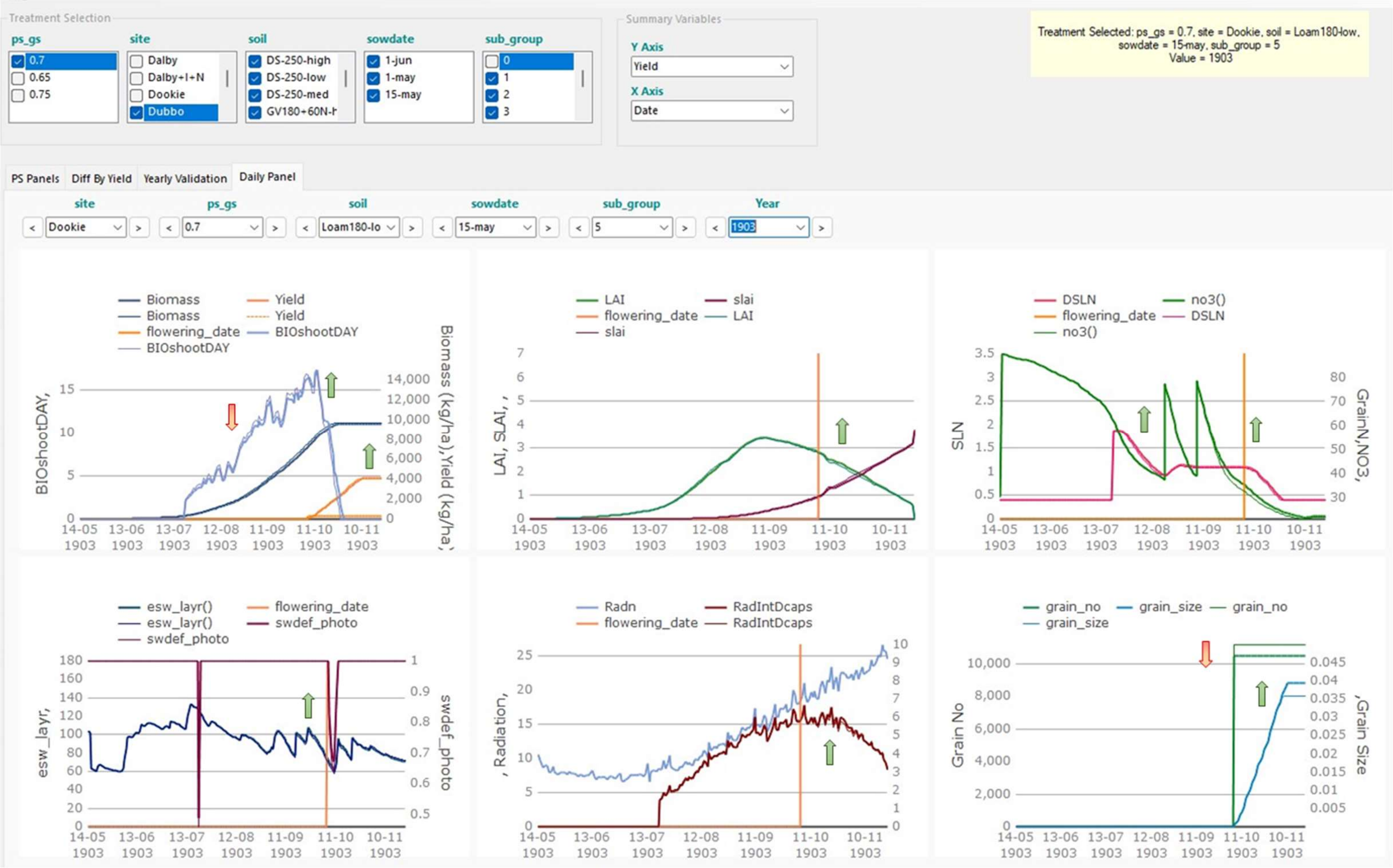

Figure S7. Wheat crop attributes trajectories with reduced growth. A season selected from the top right-hand corner of the points in Figure 5e of the main text. Baseline trajectories are thin lines; HCO3 transporter installation (target 4.1) are thick lines. Arrows highlight changes in trajectories.

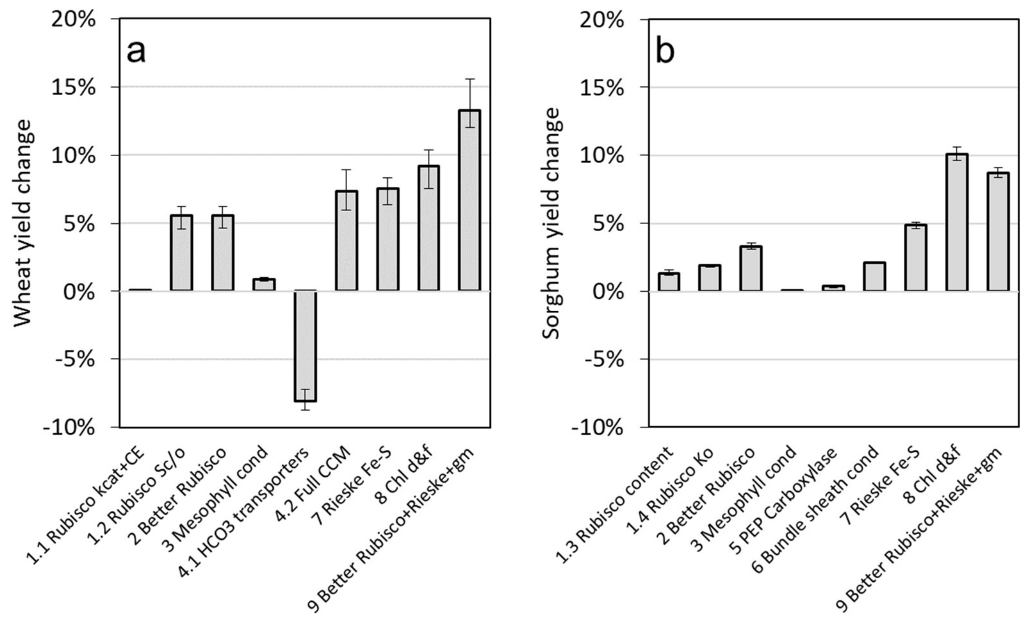

Figure S8. Simulated wheat and sorghum yield change with favourable production conditions. Crops were grown in Dalby with full irrigation and abundant N fertilisation (+I+N) (300kg/ha total). The median sowing date and starting soil water were used (Table S4). Simulated yield data from 120 seasons were used for each leaf photosynthetic manipulation (Table 1). Median values are given by bars. Whiskers show the first and third quartile values.

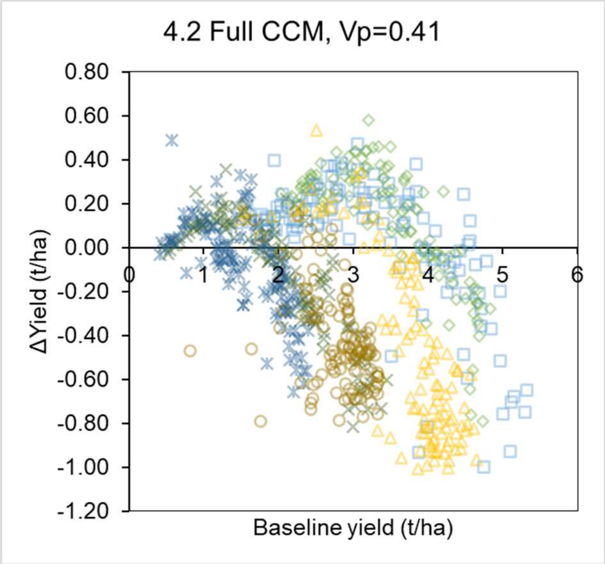

Figure S9. Analogous to Figure 5e in the main text, but with PsiVp=0.41, giving V_{bmax} of 45 instead of the C_4 sorghum level at 120. Symbols are different sites (see Figure 5).

164

Table S4. Characteristics of crop production sites, cultivars, and agronomic practices used in simulations.

Crop type	Production Region	Representative Production Site	Soil type	Soil depth (cm)	PAWC (mm)	Representative sowing dates (Early/Mid/Late)	Representative PAW (mm) at sowing (Low/Mid/High)	Standard crop configuration	Locally adapted nitrogen application (kg N ha^{-1})*
Wheat (Janz: Medium-maturing)	Queensland, northern New South Wales	Dalby (59)	Grey vertosol	180	206	1 May/ 15 May/ 1 June	172/206/206	0.25-meter row spacing and 100 plants m^{-2}	30-130-0-0
	Central, northern New South Wales	Dubbo (48)	Red dermosol	120	142		104/142/142	0.25-meter row spacing and 100 plants m^{-2}	50-50-50[d]-0
	Coastal shires in Victoria	Dookie (39)	Loam	180	109		64/109/109	0.25-meter row spacing and 150 plants m^{-2}	50-40-40[d]-40[c]
	South Australia, Victoria Southern New South Wales	Walpeup (32)	Loamy sand	130	134		31/59/113	0.25-meter row spacing and 100 plants m^{-2}	50-20-30[b]-30[c]
	North-east inland Western Australia	Merredin (14)	Shallow loamy duplex	120	101		16/37/74	0.25-meter row spacing and 100 plants m^{-2}	30-20-20-30[a]
	South-west costal Western Australia	Katanning (8)	Deep sandy duplex	250	74		46/74/74	0.25-meter row spacing and 150 plants m^{-2}	45-20-30-30[a]
Sorghum (Hybrid MR-Buster: Medium-maturing)	Central Queensland	Emerald	Black earth	120	150	15 Nov/15 Dec/15 Jan	86/98/109	1-meter row spacing and 5 plants m^{-2}	30-120-0-0
	Southern Queensland	Dalby	Vertosol	180	324	15 Oct/15 Nov/15 Dec	137/200/262		30-120-0-0
	Northern New South Wales (upper)	Moree	Grey clay	150	263		121/168/214		30-120-0-0
	Northern New South Wales (lower)	Gunnedah							50-100-0-0

*Wheat data taken from Chenu *et al.* (2013): initial and applied nitrogen (N) is indicated by '*x*-*y*-*z*-*a*': *x*, antecedent soil N (the baseline N before fertilisation) evenly present throughout soil layers at sowing; *y*, N applied at sowing at 50 mm deep as nitrate, but captured by the soil module of the simulation by adding *y* into the top layer to simplify simulation files; *z* and *a*, N applied as nitrate at the stages 'beginning of stem elongation' and 'mid-stem elongation', respectively. Sorghum data: *x* taken from Hammer *et al.* (2014); *y* calculated by recommended N application given targeting grain yield in the top quartile levels.

[a]If soil PAW > 60 mm at the stage 'mid-stem elongation".

[c]If soil PAW > 60% of PAWC at the stage 'mid-stem elongation'.

[b]If > 80 mm of rainfall from sowing to the stage 'beginning of stem elongation'.

[d]If > 100 mm of rainfall from sowing to the stage 'beginning of stem elongation'.

Table S5. Photosynthesis model parameters and their baseline values used in the C_3, single-cell CCM, and C_4 photosynthesis models. Values were adapted from Wu et al (2019) with some recalculated using new data. Values in brackets indicate level of the photosynthetic parameter in health, fully expanded leaves with a specific leaf nitrogen (SLN) of 2 g N m^{-2}.

Parameter	Units	C_3 wheat	Single-cell CCM wheat: adding bicarbonate transporters	Single-cell CCM wheat: full CCM	C_4 sorghum
χ_{Vc} (V_{cmax25})	mmol CO_2 mol^{-1} N s^{-1} (μmol m^{-2} s^{-1})	1.45 (158)	1.45 (158)		0.28 (31)
χ_{Vp} (V_{pmax25})		-	0.41 (45)*	1.1 (120)*	1.1 (120)
χ_J (J_{max25})		2.4 (271)	2.4 (271)		2.5 (277)
K_c	μbar	268	268	2656	1194
K_o	μbar	164697	164697	136698	291378
$S_{c/o}$	μbar $μbar^{-1}$	2799	2799	1713	1339
K_p	μbar	-	60**		74
χ_{gm} (g_{m25})	mol CO_2 mol^{-1} N s^{-1} bar^{-1} (mol m^{-2} s^{-1} bar^{-1})	0.005 (0.55)	0.011 (1.20^{+})		0.011 (1.20)
g_{bs}	mol m^{-2} s^{-1} bar^{-1}	-	(1^{++})	(0.003^{++})	(0.003)
φ	-	-	0.75		2
α_{PSII} (single leaf)	-	0.36	0.36		0.26
θ	-	0.7	0.7		0.68
C_i/C_a	-	0.7	0.7		0.4

* In the single-cell cyanobacterial CCM model, this value is the slope (χ_{Vb}) of the linear relationship between the maximum rate of bicarbonate transporters (V_{bmax25}) and SLN for SLN greater than a leaf structural N level.

** In the single-cell CCM model, this value is renamed the Michalis-Menten constant of bicarbonate transporter for CO_2.

$^{+}$ Conductance for CO_2 across the mesophyll cell wall plasmalemma interface

$^{++}$ Conductance for CO_2 across the chloroplast envelope.

Table S6. Long-term average cropping area, yield, and total production calculated at the regional level using shire-level data from the Australian Bureau of Statistics. Wheat values are averaged yearly values between 1901 and 2004; sorghum values are averaged yearly values between 1983 and 2015. The area and production values in the AUS Total row are consistent with the Australian Government Department of Agriculture, Water and the Environment (ABSRES) state-level crop data (file access: https://www.agriculture.gov.au/abares/research-topics/agricultural-outlook/data#australian-crop-report-data).

Crop	Region ID	Region name	Representative Production Site	Area (Mha)	Yield (t/ha)	Production (Mt)	Regional Production Weighting
Wheat	1	Queensland, northern New South Wales	Dalby	0.56	1.47	0.82	4%
	2	Central, northern New South Wales	Dubbo	2.32	1.94	4.49	24%
	3	Coastal shires in Victoria	Dookie	0.16	2.62	0.43	2%
	4	South Australia, Victoria Southern New South Wales	Walpeup	2.58	1.99	5.15	27%
	5	North-east inland Western Australia	Merredin	2.97	1.89	5.62	30%
	6	South-west costal Western Australia	Katanning	1.01	2.31	2.32	12%
	AUS Total			9.60		18.82	100%
Sorghum	1	Central Queensland	Emerald	0.18	1.73	0.32	20%
	2	Southern Queensland	Dalby	0.27	2.78	0.77	48%
	3	Northern New South Wales	Moree, Gunnedah	0.18	2.86	0.52	33%
	AUS Total			0.63		1.61	100%

Table S7. Predicted median percentage change in crop production at the regional level.

Crop	Region ID	Region name	Representative Production Site	Production (Mt)	1.1 Rubisco kcat + CE (%)	1.2 Rubisco Sc/o (%)	2 Better Rubisco (%)	3 Mesophyll cond (%)	4.1 HCO3 transporters (%)	4.2 Full CCM (%)	7 Rieske Fe-S (%)	8 Chl d&f (%)	9 Better Rubisco + Rieske + gm (%)
Wheat	1	Queensland, northern New South Wales	Dalby	0.82	1.20	-0.71	0.65	0.10	3.11	4.75	-1.58	-1.75	-0.18
	2	Central, northern New South Wales	Dubbo	4.49	1.00	-0.12	1.10	0.20	1.71	5.90	-0.89	-0.69	0.81
	3	Coastal shires in Victoria	Dookie	0.43	0.84	0.19	1.68	0.37	-0.58	3.90	-0.71	-0.18	2.07
	4	South Australia, Victoria Southern New South Wales	Walpeup	5.15	0.89	0.08	1.30	0.22	0.84	2.87	-0.42	-0.14	1.63
	5	North-east inland Western Australia	Merredin	5.62	0.93	-0.58	0.76	0.14	1.78	1.22	-1.25	-0.65	0.29
	6	South-west costal Western Australia	Katanning	2.32	0.63	0.14	1.24	0.37	0.11	1.54	-0.61	-0.40	2.04

Crop	Region ID	Region name	Representative Production Site	Production (Mt)	1.3 Rubisco content (%)	1.4 Rubisco Ko (%)	2 Better Rubisco (%)	3 Mesophyll cond (%)	5 PEP Carboxylase (%)	6 Bundle sheath cond (%)	7 Rieske Fe-S (%)	8 Chl d&f (%)	9 Better Rubisco + Rieske + gm (%)
Sorghum	1	Central Queensland	Emerald	0.32	0.87	0.95	1.86	0.09	0.44	1.16	2.14	3.87	4.13
	2	Southern Queensland	Dalby	0.77	1.33	1.00	2.39	0.05	0.59	1.34	1.95	3.62	4.66
	3	Northern New South Wales	Moree, Gunnedah	0.52	1.12	0.53	1.73	0.10	0.55	0.81	0.83	1.20	2.63

References

Chenu K., Deihimfard R. & Chapman S.C. (2013) Large-scale characterization of drought pattern: a continent-wide modelling approach applied to the Australian wheatbelt – spatial and temporal trends. *New Phytologist*, **198**, 801-820.

Farquhar G.D. (1983) On the nature of carbon isotope discrimination in C4 species. *Australian Journal of Plant Physiology*, **10**, 205-226.

Farquhar G.D., von Caemmerer S. & Berry J.A. (1980) A biochemical model of photosynthetic CO2 assimilation in leaves of C3 species. *Planta*, **149**, 78-90.

Farquhar G.D. & Wong S.C. (1984) An empirical-model of stomatal conductance. *Australian Journal of Plant Physiology*, **11**, 191-209.

Hammer G.L., McLean G., Chapman S., Zheng B., Doherty A., Harrison M.T.... (2014) Crop design for specific adaptation in variable dryland production environments. *Crop and Pasture Science*, **65**, 614-626.

Maxwell K. & Johnson G.N. (2000) Chlorophyll fluorescence—a practical guide. *Journal of Experimental Botany*, **51**, 659-668.

Price G.D., Badger M.R. & von Caemmerer S. (2011) The Prospect of Using Cyanobacterial Bicarbonate Transporters to Improve Leaf Photosynthesis in C3 Crop Plants. *Plant Physiology*, **155**, 20-26.

von Caemmerer S. (2000) *Biochemical models of leaf photosynthesis. Vol. 2*. CSIRO Publishing, Collingwood, Australia.

von Caemmerer S. (2021) Updating the steady-state model of C4 photosynthesis. *Journal of Experimental Botany*, **72**, 6003-6017.

Yin X. & Struik P.C. (2012) Mathematical review of the energy transduction stoichiometries of C4 leaf photosynthesis under limiting light. *Plant, Cell & Environment*, **35**, 1299-1312.
